# Analysis of amplification and association polymorphisms in the bovine beta-defensin 129 (BBD129) gene revealed its function in bull fertility

**DOI:** 10.1038/s41598-022-23654-3

**Published:** 2022-11-09

**Authors:** Subhash Solanki, Poonam Kashyap, Syed Azmal Ali, Vijay Kumar, Ashutosh Vats, Martina Pukhrambam, Rakesh Kumar, Sachinandan De, Tirtha Kumar Datta

**Affiliations:** 1 Animal Genomics Lab, National Dairy Research Institute, Karnal, Haryana 132001 India; 2 Cell Biology and Proteomics Lab, Animal Biotechnology Centre, National Dairy Research Institute, Karnal, 132001 Haryana India; 3 NMR-2 Lab, National Institute of Immunology, New Delhi, 110076 India; 4 Present Address: ICAR- Central Institute for Research on Buffaloes, Hisar, 125001 India

**Keywords:** Biotechnology, Animal biotechnology, Genomics, Sequencing

## Abstract

β-defensins are adsorbable on the sperm surface in the male reproductive tract (MRT) and enhance sperm functional characteristics. The beta-defensin 129 (*DEFB129*) antimicrobial peptide is involved in sperm maturation, motility, and fertilization. However, its role in bovine fertility has not been well investigated. This study examines the relationship between the bovine *BBD129* gene and *Bos indicus x Bos taurus* bull fertility. The complete coding sequence of *BBD129* mRNA was identified by RNA Ligase Mediated-Rapid Amplification of cDNA End (RLM-RACE) and Sanger sequencing methodologies. It consisted of 582 nucleotides (nts) including 5' untranslated region (UTR) (46nts) and 3'UTR (23nts). It conserves all beta-defensin-like features. The expression level of BBD129 was checked by RT-qPCR and maximal expression was detected in the corpus—epididymis region compared to other parts of MRT. Polymorphism in BBD129 was also confirmed by Sanger sequencing of 254 clones from 5 high fertile (HF) and 6 low fertile (LF) bulls at two positions, 169 T > G and 329A > G, which change the S57A and N110S in the protein sequence respectively. These two mutations give rise to four types of BBD129 haplotypes. The non-mutated TA-*BBD129* (169 T/329A) haplotype was substantially more prevalent among high-fertile bulls *(P* < 0.005), while the double-site mutated GG-*BBD129* (169 T > G/329A > G) haplotype was significantly more prevalent among low-fertile bulls (*P* < 0.005). The in silico analysis confirmed that the polymorphism in BBD129 results in changes in mRNA secondary structure, protein conformations, protein stability, extracellular-surface availability, post-translational modifications (O-glycosylation and phosphorylation), and affects antibacterial and immunomodulatory capabilities. In conclusion, the mRNA expression of *BBD129* in the MRT indicates its region-specific dynamics in sperm maturation. *BBD129* polymorphisms were identified as the deciding elements accountable for the changed proteins with impaired functionality, contributing to cross-bred bulls' poor fertility.

## Introduction

Reproduction's molecular mechanism is a complicated process involving hundreds of genes. Any abnormalities or polymorphisms in these genes impact the processes of gametogenesis, sperm transit to the egg, fertilization, and embryo creation, either directly or indirectly^1–3^. In the last decade, breakthroughs in molecular omics technologies, i.e., genomics, transcriptomics, and proteomics, have increased the discovery of linked reproductive indicators for choosing the finest breeding domestic animals^4–7^. The testicular sperm are non-motile and unable to fertilize the egg; thus, the maturation of sperm occurs in the epididymis, where sperm are exposed to a complex of chemicals secreted by epididymal epithelial cells^8,9^. These uptakes of epididymal glycoproteins on the sperm surface protect sperm from adverse events such as premature capacitation acrosomal reaction, provide motility, and aid sperm in traversing the hostile female reproductive tract (FRT) barriers, such as cervical mucus passage, uterine immune evasion, oviduct epithelial cells (OECs) binding, and zona interactions^2,8,10–13^.

During epididymal maturation, the presence of an essential class of glycosylated beta-defensin proteins is one of the most important sperm surface changes^9^. β-defensin (BD) is a most ancient class of defensin, and it is conserved in disulfide linkages viz. C1-C5, C2-C4, and C3-C6^14–16^ give rise to the typical BD motif with a defensin fold containing three antiparallel beta-strands^17–19^. Most known BD genes have two exons and conserved protein signal sequences. The signal peptide is encoded by the first exon and cleaved off to produce the mature functional peptide encoded by the second exon. The exposure of amino acids on the surface of functional proteins is conserved at cysteine locations, indicating the stability of the core configuration of protein structure for their conserved antimicrobial properties over the development of species-specific adaptation^20–22^. Even though defensins were initially complete, BD genes have undergone duplications and non-synonymous mutations over evolution, giving rise to different clusters with region-specific roles and acquiring their specialized reproductive functions^14,23^.

Class-A BDs, such as beta-defensin 126 (*DEF126*) and *DEFB12/BBD129*, have shown age- and sex-specific expressions indicating their pleiotropic physiological importance in humans^24^, macaques^10,11,25^, rodent^26,27^, bovines^28,29^, owine^18^, and equine^30^. In primates, the highly glycosylated *DEFB126* and rat *DEFB22* (orthologs) are coated on the surface of sperm and promote sperm activity. Additional release of these BDs during capacitation is necessary for sperm-zone interactions^25^. Polymorphisms in the BD genes result in lower milk content^31,32^, increased round cells in sperm^33^, male fertility^34^, impaired sperm capacity to penetrate mucus^2,35^, OECs binding^10,36^, and egg contact^37^. Adding recombinant BDs (r-BDs) to defective spermatozoa enhanced their antibacterial activity, motility^37–39^, OECs, and zona binding capacities^10,25^. The buffalo beta-defensin 129 (*BuBD129*) gene has emerged as a new bovine ortholog of primate *DEFB126* with greater expression than other members of the class-A beta-defensins (CA-BDs) family^15,23^. Rat *DEFB129* or human *DEFB116* produced in the epididymis binds the sperm surface chemokine receptor *CCR6* and promotes sperm motility via modulating calcium ion influx^39^. However, no information is available in connection to *BBD129* (*Bos taurus*) polymorphisms to bulls' reproductive and fertility performance.

Despite their significance, the genomic characterization of most β-defensin genes in all species, including bovines, remains incomplete and speculative. Keeping in mind the significance of CA-BDs in male reproduction, the current research work sought to establish and describe the complete mRNA architecture of the *BBD129* gene, including the untranslated regions (UTRs) and its expression dynamics in the male reproductive tract. Finally, examine the correlations between genomic polymorphisms of the *BBD129* gene and the fertility of cross-bred bulls.

## Materials and methods

### Male reproductive tissue sample collection and BBD129 RLM-RACE assay

#### Sample collection and processing

The matured male reproductive tract (MRT) of Indian cross-bred cattle (*Bos indicus x Bos taurus*) (n = 3 biological replicates, and age 3–5 years old matured bulls) were collected from the Kolkata cattle abattoir, Kolkata, India (Animal ethics approval IAEC No. 144/16). The MRT samples were washed with 1X PBS saline buffer solution, and to maintain RNA quality, samples were frozen in the dry ice within 10–15 min of slaughter. In the laboratory, the MRT tissues viz. Rete testes (RT), seminiferous tubule (ST), caput, corpus, cauda, and vas-deferens (VD) were dissected into 3 to 5 mm size pieces under sterile conditions (Supplementary Fig. 1).

#### RNA extraction and DNase I treatment

Based on prior literature information and our lab data, the caudal region was selected for total RNA isolation. The caudal tissue was frozen into liquid nitrogen gas and crushed into mortar pestle followed TRIzol methodology (Qiazol, USA)^23,25^. The DNase-I enzyme treatment was done as per company manufacture protocol (#18,047,019, ThermoFisher Scientific, Waltham, Massachusetts, USA. RNA quantity and quality was measured by NanoDrop ND-1000 spectrophotometer (Thermo NanoDrop Technologies, USA) (Supplementary Fig. 2) and 1% agarose gel electrophoresis (AGE).

#### RLM-RACE primer designing strategy, cDNA synthesis and RACE PCR

To amplify the 5' and 3' ends of the *BBD129* mRNA, two sets of nested primers were designed as suggested by the RLM-RACE procedure^40^. The *BBD129* mRNA sequence of *Bos taurus* cattle was retrieved from the ENSEMBL database (transcript ID: *DEFB129*-201 ENSBTAT00000064705.2), NCBI. All primers were designed by NCBI primer-Blast tool^41^. The primer parameters were checked and confirmed by the Oligocal server^42^. All the primers used in this study are provided in supplementary table 1.

#### RLM-RACE of BBD129

The 5' and 3' ends of the *BBD129* mRNA were amplified by the RLM-RACE kit (#AM1700M, Invitrogen, Waltham, Massachusetts, USA) as per company suggested protocol^43^. The RNA processing steps for the 5' RLM-RACE were following:A) *Calf Intestine Alkaline Phosphate (CIP) treatment-Dephosphorylation of incomplete and non-mRNA,* B) *Termination of CIP enzyme*, C) *Tobacco Acid Pyrophosphatase (TAP) associated enzymatic decapping of mRNA molecules,* and D) *5' RLM-RACE Adapter ligation*^43,44^.

#### Reverse transcription for the cDNA synthesis

It was performed using the SuperScript™ IV First-Strand Synthesis kit (#18,091,050, ThermoFisher Scientific, USA) per the company's manufacturer's protocol. The 5’ adapter-ligated mRNA (10 µl) was used as a template for cDNA synthesis. Negative controls of *Tobacco Acid Pyrophosphatase* control (-*TAP*) and non-template control (NTC) were run to check cDNA synthesis from incomplete or degraded mRNA and genomic DNA contamination, respectively. In brief, the 200 ng of the 5' adapter-ligated RNA template was mixed with random hexamer & oligo_(dT)n primers (1 µl each) and DEPC-treated water into a sterile PCR tube followed by incubation at 65 °C for 5 min and then immediate ice incubation for 2 min. After that, the 5X SuperScript IV reaction buffer, RNase inhibitor, dNTP mix, and RNase inhibitor, *SuperScript IV reverse transcriptase* were added to make a final 20 μl PCR reaction. The reaction was conducted at 55 °C for 30 min in a PCR thermal cycler machine. The reverse transcriptase was inactivated by heating at 90 °C for 10 min.

#### The 5' RLM-RACE PCR:

The 5' RLM-RACE nested PCR was performed as per kit protocol by using primers sets mentioned in supplementary table 1^44^. For outer 5’ RACE PCR, a 50 µl volume of PCR reaction consisted of the following: the 5' adapter-ligated cDNA, 5X Phusion HF reaction buffer, dNTPs, primers, high fidelity *Phusion DNA Taq polymerase*, and nuclease-free water. The PCR thermal profile was: initial denaturation at 98 °C for 30 s, denaturation at 98 °C for 30 s, annealing at 63 °C for 30 s, and extension at 72 °C for 45 s. A total of 35 cycles of the PCR were performed, with the last extension of 10 min at 72 °C. Two negative control reactions, viz. negative TAP and non-template reaction, were run to confirm full-length RNA integrity and DNA contamination, respectively. For inner 5’ RACE PCR, a 2 µl of 1000 time dilution of 5’ RACE PCR product was used with similar PCR reaction compositions and thermal profile. The amplified products were run on 2% AGE.

#### The 3' RACE PCR:

As mentioned above, a fresh total RNA extraction was done from the caudal region, and cDNA synthesis was performed. The 3' RACE adapter (5'-GCGAGCACAGAATTAATACGACTCACTATAGGT12VN-3') was used as a primer in the reverse transcription reaction with all the similar conditions as done above. The 3' RACE nested PCR was performed using 3’ end’s outer and inner gene-specific primer sets (supplementary table 1). The outer and inner 3' RACE PCR reaction compositions and PCR thermal profiles were similar to the 5' RACE PCR. A 1000-time dilution of 3’ outer RACE PCR was used as the nucleic acid template for 3’ RACE PCR. We have also run an internal *BBD129* cDNA PCR with cDNA-specific primers with the same PCR reaction composition and thermal profile as mentioned in the 5' RACE PCR to amplify the internal coding region of *BBD129* mRNA. The amplified products were run on 2% AGE.

#### Cloning and sequencing of RLM-RACE amplified products

*Cloning:* The cloning was done using the CloneJET 1.2 kit per the company-manufactured protocol (#K1231, ThermoFisher Scientific, Waltham, Massachusetts, USA). A ligation reaction of 20 µl volumes consisted of pJET1.2 blunt cloning vector, *T4 DNA ligase*, *T4 DNA ligase* buffer, PCR eluted 3' & 5' RLM-RACE products, and nuclease-free water. The ligation reaction was incubated at room temperature (RT) for 1 h and subsequently used to transform to competent XL-1 blue E.coli bacteria at 48 °C for 90 s in a pre-warmed water bath. The LB agarose medium containing ampicillin (50 µg/ml) was used to check transformation. The positive clones were picked from the agarose plate and confirmed by colony PCR. The colony PCR reaction composition and thermal profile were similar, as mentioned in the 5' RACE PCR.

#### Plasmid isolation and plasmid PCR

The positively transformed colonies were inoculated into LB broth medium, and a 2 ml of overnight grown transformed bacterial broth culture was centrifuged at 8000 g at RT for 10 min. The pellet was dissolved in alkaline lysis solution-1 (50 mM glucose, 25 mM Tris–HCl, 10 mM EDTA), alkaline lysis solution-2 (0.2 N NaOH, 1 percent SDS), and alkaline lysis solution-3 (5 M potassium acetate, glacial acetic acid) and incubated for 10 min at RT followed by the alkaline lysis method of plasmid isolation^45^.The plasmid quantity and quality was evaluated by NanoDrop ND1000 spectrophotometer and AGE.

*Plasmid PCR: *Before sequencing, plasmid PCR was done to confirm the persistence of the 5’ and 3’ ends amplified r-*BBD129* fragments in the plasmids. The positive plasmids were sent for sequencing.

### The BBD129 mRNA characterization and phylogenetic analysis

#### Genomic characterization

The sequencing results of *BBD129* RLM-RACE PCR products were BLAST (BLASTn and BLASTp) against cattle genome (*Bos indicus x Bos taurus* ) on NCBI (UOA_Brahman_1 GCF_003369695.1) and USSC-Genome Browser (Apr. 2018 ARS-UCD1.2/bosTau9)^46^.

The BD like characteristics such as *Cysteine bond pairing prediction, Secondary structure prediction, Signal peptide prediction, and Biological function prediction* were made by *the DiANNA* Web server^47^, *PSIPRED* and *SOPMA* web servers^48,49^, *Signal IP 5.0* server^50^, and the *Argot2* server^51^, respectively. The complete sequence of cattle *BBD129* gene was submitted on NCBI-BankIt^52^.

#### Evolutionary phylogenetic tree analysis

The protein sequences of the *DEFB129* gene from different ruminant and non-ruminant mammalian species were retrieved from the NCBI database using the keyword "beta-defensin 129" (supplementary file 1). Evolutionary phylogenetic analysis was performed using the MEGA X bioinformatics tool and Maximum Likelihood method, and Tamura-Nei model to find out the evolutionary relationships between RLM-RACE identified cross-bred cattle *BBD129* (Bovidae family) and its neighbors family in other species^53^. This analysis involved 29 protein sequences of the *DEFB129* gene from different species. The bootstrap consensus tree inferred from 1000 replicates was taken to represent the evolutionary history of the taxa analyzed.

### RT-qPCR expression analysis

#### RNA isolation, optimization and RT-qPCR assay

As mentioned above, total RNA extraction from frozen cattle MRT tissues and cDNA synthesis were extracted from the samples. The primers were designed from our RLM-RACE *BBD129* product (supplementary table 1). The *GAPDH* (*glyceraldehyde-3-phosphate dehydrogenase*) and *eEF-2 *(*eukaryotic elongation factor 2*) genes were chosen as reference^18,54^.

The *Optimization of RT-qPCR parameters* such as annealing temperature (*Tm)*and *Primer concentration optimizations* were done as previously in our lab^23^.

*The relative expression* of the cattle *BBD129* gene was done in duplicate in a 10 µl RT-qPCR reaction volume containing a 5 µl iTaq Universal SYBR Green Supermix (#1,725,129, BioRad, Hercules, California, USA), 1 µl each primer and nuclease-free water. The RT-qPCR thermal profile was: initial denaturation (95 °C for 3 min), denaturation (95 °C for 10 s), annealing (49 °C for 15 s), extension (72 °C for 15 min), a total of 35 cycles were run on Bio-Rad CFX96 Maestro qPCR machine (Bio-Rad, USA)^23^. To ensure specificity and any primer-dimer formation, we performed a melting peak analysis with a temperature gradient of 0.5 °C/sec from 65 °C to 95 °C at the end of amplification. A non-template control was run to cross-confirm nucleic acid contamination with PCR components. RT-qPCR efficiency was calculated according to the E = 10(-1/slope)^55^.The MIQE guidelines were followed at each step of RT-qPCR and RT-qPCR data analysis to ensure the quality.

### Genomic amplification of the BBD129 gene

#### Collection of frozen semen samples and sperm genomic DNA extraction

*Collection of Frozen samples: *A total of eleven distinct fertility cross-bred bulls were selected based on percentages of their first and second artificial insemination conception rate (CR) data available at the Artificial Breeding Research Center, National Dairy Research Institute, India. The bulls with a CR of less than 31.79% were classified into the low fertile (LF) group, and the bulls with a CR of more than 50.57% were classified into the high fertile (HF) group (Table 1). The frozen semen from five HF bulls and six LF bulls were collected into liquid nitrogen and shifted to the lab.

**Table 1:** The selection of high fertile and low fertile cross-bred bulls was done on the basis of percentage of their conception rate data available on the NDRI herd.

Cattle bulls No	Conception rate (%)	Number of BBD129 clones	BBD129 TA Haplotype% (169 T/329A)	BBD129 GG Haplotype % (169G/329G)	BBD129 GA Haplotype % (169G)	BBD129 TG Haplotype % (329G)	Remarks
HF-1	50.75	18	72.22%	16.66%	0%	11.11%	Total clone sequenced = 105 TA haplotype = 72.21% GG haplotype = 23.16% GA haplotype = 2.2% GA haplotype = 2.2%
HF-2	55.13	23	65.21%	34.78%	0%	0%	
HF-3	54.65	17	82.35%	17.64%	0%	0%	
HF-4	57.30	25	64%	24%	12%	0%	
HF-5	57.81	22	77.27%	22.72%	0%	0%	
LF-1	29.52	22	13.63%	86.36%	0%	0%	Total clone sequenced = 149 TA haplotype = 29.18% GG haplotype = 68.65% TG haplotype = 2.68%
LF-2	30.17	22	45.45%	45.45%	0%	9.09%	
LF-3	28.36	21	38.09%	61.90%	0%	0%	
LF-4	27.19	19	0%	100%	0%	0%	
LF-5	31.79	19	10.52%	89.48%	0%	0%	
LF-6	30.85	46	67.39%	28.26%	0%	4.34%	

The distributions of the percentage of different haplotypes of *the BBD129* gene in the distinct fertility bulls were calculated from sequenced clones.

HF , high fertile bull; LF ,  low fertile bull; Nucleotide polymorphism: G = Guanine, A = Adenine, T = Thymine, TA = 169th T & 329th A; GG = 169th G & 329th G; GA = 169th G & 329th A; TG = 169th T & 329th G; HF , High fertile; LF ,  Low fertile.

#### Genomic DNA extraction

Two frozen semen straws from each bull were thawed into the pre-warmed distilled water (37 ˚C) for 30 s, and then the contents were collected into a 15-ml falcon tube containing an 8 ml of washing buffer-A (150 mM NaCl and 10 mM EDTA) and followed by centrifugation at 800 g for 10 min. The pellet was dissolved into 300 µl of lysis buffer-B (100 mM TRIS–Cl, 10 mM EDTA, 0.5 M NaCl, and 1% SDS), 100 µl of 1 M DTT (Dithiothreitol), and 100 µl of Proteinase-K enzyme (0.2 mg/ml) and followed by overnight incubation at 55 ºC in a dry bath. The following day, the sperm gDNA was isolated by the Phenol: Chloroform: Isoamyl alcohol (PCI) method^56^. The quantity and quality of gDNA were analyzed by NanoDrop ND1000 spectrophotometer and 1% AGE.

#### BBD129 genomic DNA amplification and cloning

The exons of the *BBD129* gene were amplified by designing the primers from the exon flanking intron region (NCBI Accession: XM_027558171.1) (Table 1). The following PCR compositions were used to amplify both exons in a separate 50 µl volume reaction: 2 µl of each primer set for exon first and exon second (Table 1), 1 µl dNTPs, 10 µl Phusion buffer, 0.5 µl Phusion high fidelity DNA Taq polymerase, 1 µl gDNA template, and nuclease-free water. The following PCR thermal profile was used to amplify both exons of *BBD129*: initial denaturation (98 °C for 5 min), denaturation (98 °C for 30 s), annealing (58.2 °C for *BBD129* exon first for 30 s and 60 °C for *BBD129* exon second for 30 s), and polymerization (72 °C for 40 s). A total of 35 cycles were run, with an extension at 72 °C for 10 min at the end of amplification. *Bacterial cloning and colony PCR* was done similarly as mentioned in the RLM-RACE strategy with slight changes in primers and annealing temperature (58.2 °C for *BBD129* exon first and 60 °C for *BBD129* exon second). Plasmid isolation and plasmid PCR were done similarly, as mentioned in the *BBD129* RLM-RACE strategy. PCR positive clones were sent for the sequencing.

### Bioinformatics analyses of the BBD129 polymorphism

The bioinformatics means of observed nsSNPs in the BBD129 gene were found by the following tools:

#### NCBI BLAST alignment

BLASTn and BLASTp alignment analysis was used for the nucleotide and protein search alignment against the bovine genome, respectively^57^.

#### Prediction of functional impacts of nsSNPs on BBD129 mRNA secondary structure

The native *BBD129* (non-mutated) and double mutated mRNA sequences were submitted to the RNAfold server^57^. It provides three structural information: i) conventional secondary structure graph, ii) Dot plot analysis, and iii) Mountain plot analysis. An mRNA mountain plot is an x–y graph and shows the minimum free energy structural curve, centroid structural curve, partition function curve and positional entropy analysis resulting from the nucleotide pair probabilities.

#### Prediction of functional impacts of nsSNPs on BBD129 protein stability and functionalities

*PROVEN* (Protein Variation Effect Analyzer)^58^, *SIFT* (Sorting Intolerant from Tolerant)^59^, *Polyphen-I* (Polymorphism Phenotyping-I) & *Polyphen-II*^29^, *SNPs & GO*^60^, and *PhD-SNP* ^61^bio-computational tools were used to predict the impacts of amino acid substitutions (AAS) (viz. S57A & N110S) on the *BBD129* protein structural stability information and functions. *Polyphen* algorithm score denotes substitution benign if the score is < 0.4; possibly damaging (0.4–0.9), and probably damaging (> 0.9). *SIFT* alignment score denotes deleterious (< 0.05) and neutral (> 0.05). *PROVEN* algorithm score denotes deleterious (< − 2.5) and neutral (> − 2.5)^62^; *screening for non-acceptable polymorphisms 2* (*SNAP2*) server predicts the disease-related variants^63^; *I-Mutant 2.0* predict protein stability depending on support vector machine algorithm, and *ProTherm* server helped to evaluate AAS free energy changes values (DDG = DGmutant – DG wild type)^61,64^; *MAPP* (multivariate analysis of protein polymorphisms) was used for the interpretation of missense variant causing deleterious effect^65^. *PSIPRED* and *SOPMA* servers were used to predict the impacts of nsSNPs on protein secondary structure.

#### Prediction of impacts of snSNPs on protein physicochemical properties and post-translational modifications

The biological function prediction was done by the *Argot2* server^51^. *ProtParam* and *Mupro* bioinformatics tools were used to compute native and mutated *BBD129* protein physicochemical properties^64,66^. Post-translational modification predictions: (1) O-glycosylation site prediction was done using *NetOGlyc 4* server and *GlycoEP* standard predictor under the composition profile of pattern^67,68^. (2) N-glycosylation site prediction was done using *the NetNGlyc1.0* server^69^. (3) Phosphorylation site prediction was done using *NetPhos 3.1* server^70^. The translated amino acid sequences of the non-mutated and double site mutated *BBD129* gene were submitted above servers.

### Statistical analysis

A GraphPad Prism 5.0 unpaired *t-test* was used to analyze cattle *BBD129* gene haplotype frequency percentage distributions in the HF and LF bulls^71^. The data was normalized by data percentage before analysis. We did descriptive statistics analysis (Kurtosis and Skewness) to check the normal distribution of SNPs. To compare SNPs data of high fertile with low fertile bulls, the unequal sample size of high fertile was normalized by geometric mean before t-test analysis (unpaired t-test with Welch's correction). A *P*-value < 0.05 was set to be statistically significant. The geometrical mean of Cq (cycle of quantification) values for cattle *BBD129* transcript in triplicate samples and the relative expression ratio of *BBD129* gene expression were computed using the 2-∆∆ct method. The GraphPad Prism 5.0 software implements the Ct (threshold cycle) method^72^ and graph. *GAPDH* and *eEF2* genes were used as reference genes in the mRNA expression experiment. The *BBD129* gene expression in the seminiferous tubule (ST) region was used to normalize Cq values obtained from other MRT regions. The differential *BBD129* gene expression level was analyzed by One-Way ANOVA (Tukey post-hoc correction test) in GraphPad Prism 5.0.

### Ethics declarations

All the material and methods, we used in our study were preapproved from NDRI’s Institutional animal ethics committee under approval IAEC No. 144/16 on 3*rd* Dec, 2016.

## Results

### Complete coding BBD129 gene in Indian cross-bred cattle

**Figure 1:**
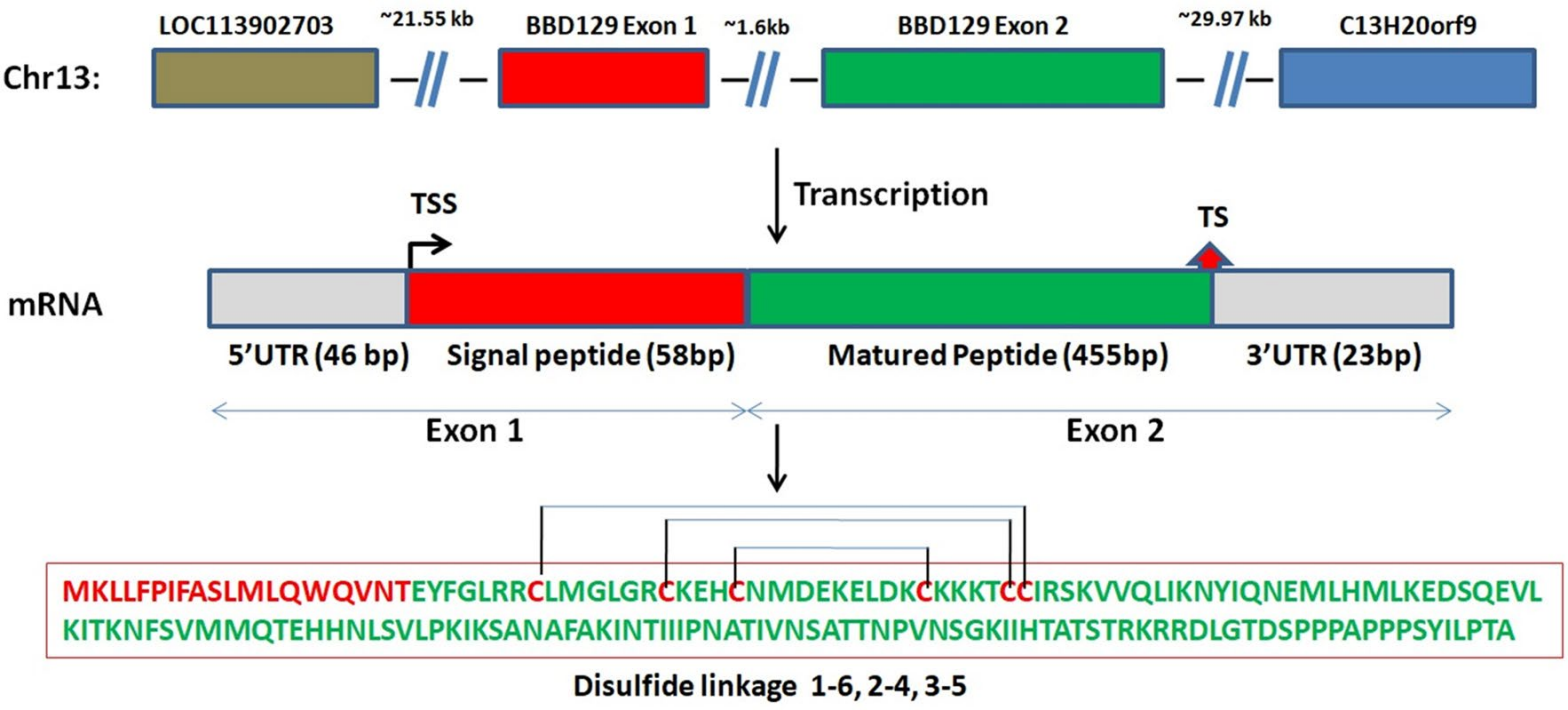
The full-length coding *BBD129* mRNA characterization in *Bos indicus x Bos taurus*. The *BBD129* gene is located on chromosome 13, and it encodes for 582 bp long mRNA transcript, giving a 171 amino acids long polypeptide. The DiANNA web server has predicted Cys1-Cys6, Cys2-Cys4 and Cys3-Cys5 disulfide linkages. Abbreviations: Chr = Chromosome, TSS = Transcription start site, TS = Termination site, and UTRs = Untranslated regions.

The 5' RLM-RACE inner PCR had shown a band of approximately 190 base pair (bp), and the 3' RACE PCR had shown an approximately 300 bp long amplified product on agarose gel (Supplementary Fig. 3). The PCR product sequences of the 5' end of *BBD129*, 3' end of *BBD129*, and internal cDNA were aligned to generate a complete coding sequence of *BBD129* mRNA (Supplementary file 2). The *BBD129* gene was located on chromosome 13, i.e., chr13: 22,660,780–22,799,850 (NCBI: UOA_Brahman_1 GCF_003369695.1) USSC: Apr. 2018 ARS-UCD1.2/bosTau9) flanked by the *LOC11390270* gene upstream and the *C13H20orf96* gene in the downstream region. The analysis found that *the BBD129* gene consisted of two exons separated by a non-coding intron region of approximately l.6 kb. The first exon codes for the 46 bp long 5' UTR region and signal peptide (58 bp), while the second exon codes for the functional protein part (455 bp) and a 23 bp long 3' UTR region (Fig. 1). Gene ORF starts at the 47th nucleotide and ends with the 560^th^ nucleotide, carrying a 511-nucleotide long coding mRNA. Exon one shared its last nucleotide (58^th^) with the second exon to produce a 171-amino-acid long peptide. The complete length of the *BBD129* mRNA sequence (*Bos indicus x Bos taurus) *was submitted to the NCBI with an accession number MW900256 (BankIt number: 2449612) (Fig. 1 and supplementary table 1). The *BBD129* gene conserves its BD-like characteristics such as cysteine amino acid pairing (disulfide bonding), a general characteristic of the BD family, i.e., C1-C5, C2-C4, and C3-C6, and protein secondary structure with a conserved N-terminal signal peptide of 19 amino acids. The *BBD129* protein bears a protease cleavage site between amino acids 19 and 20, which has been predicted with a cleavage probability of 0.998 (supplementary Fig. 4). The Argot2 predicted that it is involved in many biological functions such as immune defensive roles, efficient binding with *CCR6* chemokine receptor and lipopolysaccharide-binding. The C-terminal amino acids have extensive efficiency of post-translation modifications such as O-glycosylations and phosphorylations (Fig. 1 and supplementary table 1).

### Phylogenetic tree analysis:

**Figure 2:**
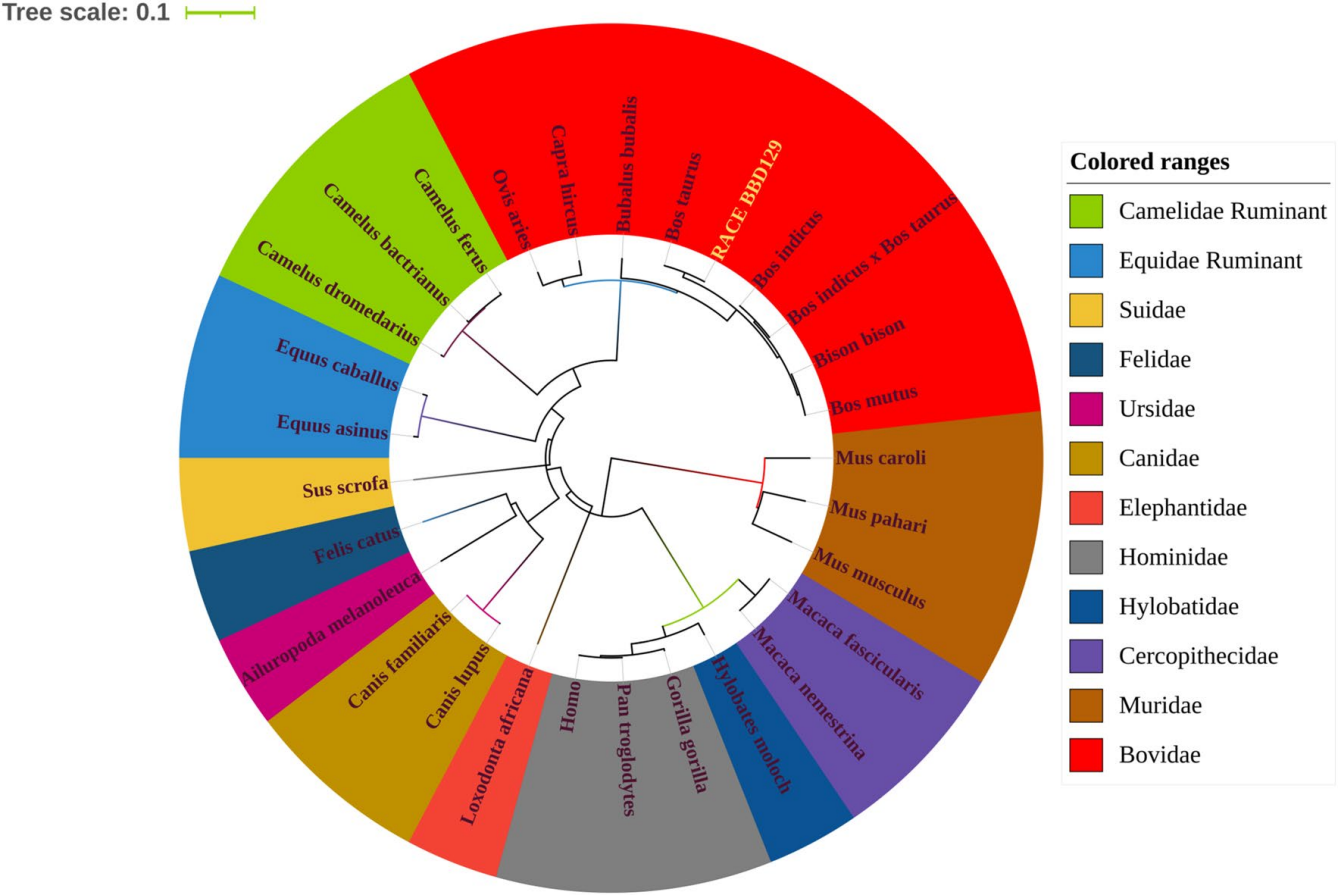
The *BBD129* gene phylogenetic tree analysis was performed using the MEGA-X bioinformatics tool. The RLM-RACE amplified *BBD129* mRNA has shown a strong evolutionary relationship within the Bovidae family members. The *BBD129* amino acid sequence variations increase as the distance between mammalian species increases.

The multiple sequence alignment (MSA) and phylogenetic analyses of the *BBD129* gene across twenty-nine mammalian species indicated a strong evolutionary relationship between the RLM-RACE amplified*BBD129* gene of *Bos indicus x Bos taurus* and the Bovidae family (Fig. 2; supplementary Fig. 5). The Camelidae and Equidae families had shown relationships next to the Bovidae family. The MSA analysis has found Indian cattle *BBD129* gene is structurally similar to *Bos taurus* except for a few amino acids.

### Expression of beta-defensin BBD129 gene in the male reproductive tract

**Figure 3:**
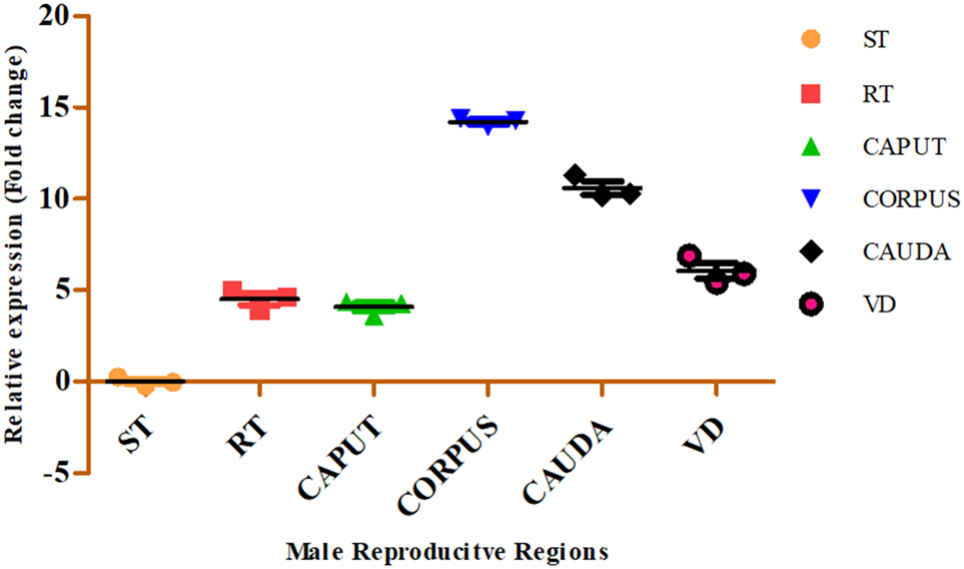
RT-qPCR expression analysis of *BBD129 mRNA* shows a dynamic pattern throughout the male reproductive tract. About 14 folds of *BBD129* mRNA were observed in the corpus-epididymis region compared to the normalizer region, viz. seminiferous tubule (ST). The lowest expression (~ 4 folds) of *BBD129* mRNA was observed in the caput-epididymis region.

The RT-qPCR analysis of *BBD129* in the MRT of adult *Bos indicus x Bos taurus* cattle has shown that the expression dynamics of *BBD129* were slightly different from the other mammalian species. The higher expression of *BBD129* was observed in the corpus segment of the epididymis compared to other MRT tissues (viz. ST, RT, caput, cauda, and VD) (Fig. 3). The corpus region has shown about 14.2 fold higher expression than the normalizer ST region, followed by the cauda region. In comparison to the ST region, the expression of the *BBD129* gene in other MRT parts was: rete testis (4.5 fold), caput-epididymis (4.0 fold), cauda (10.58 fold), and VD (6.0 fold). The caput region was observed to have the lowest expression of *BBD129* mRNA, and then it was shown to be higher in the corpus region, and after that, it declined. Still, it has maintained the expression till the sperm ejaculate via the VD region. The details of mean difference and *P*-values have been provided in supplementary table 1.

### Association of cattle BBD129 polymorphisms with bull fertility

**Figure 4:**
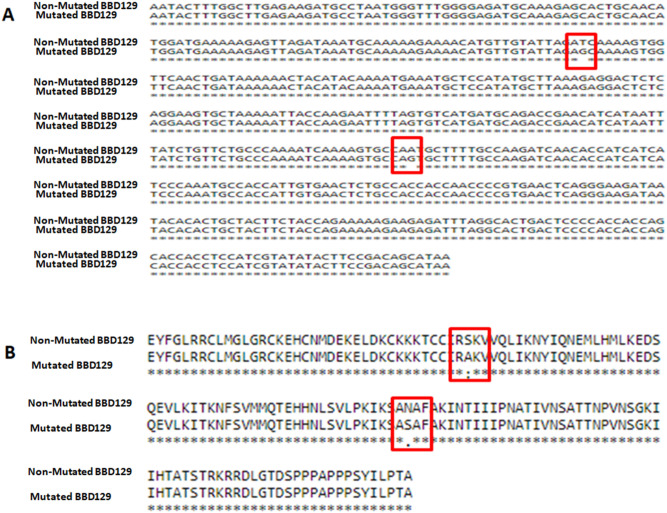
NCBI-BLAST of genomic DNA amplified *BBD129* gene from distinct fertility cross-bred cattle bulls. (**A**) The sequence variations were observed at the nucleotide level in the in the sequenced clones. Two polymorphisms were found in the exon-2 at 169th (T/G) and 329th (A/G) nucleotide positions. (**B**) The variations were also observed at the amino acids level. The *BBD129* gene has shown two amino acid substitutions i.e., one at the 57th position (Serine to Alanine) and the 110th position (Asparagine to Serine).

**Figure 5:**
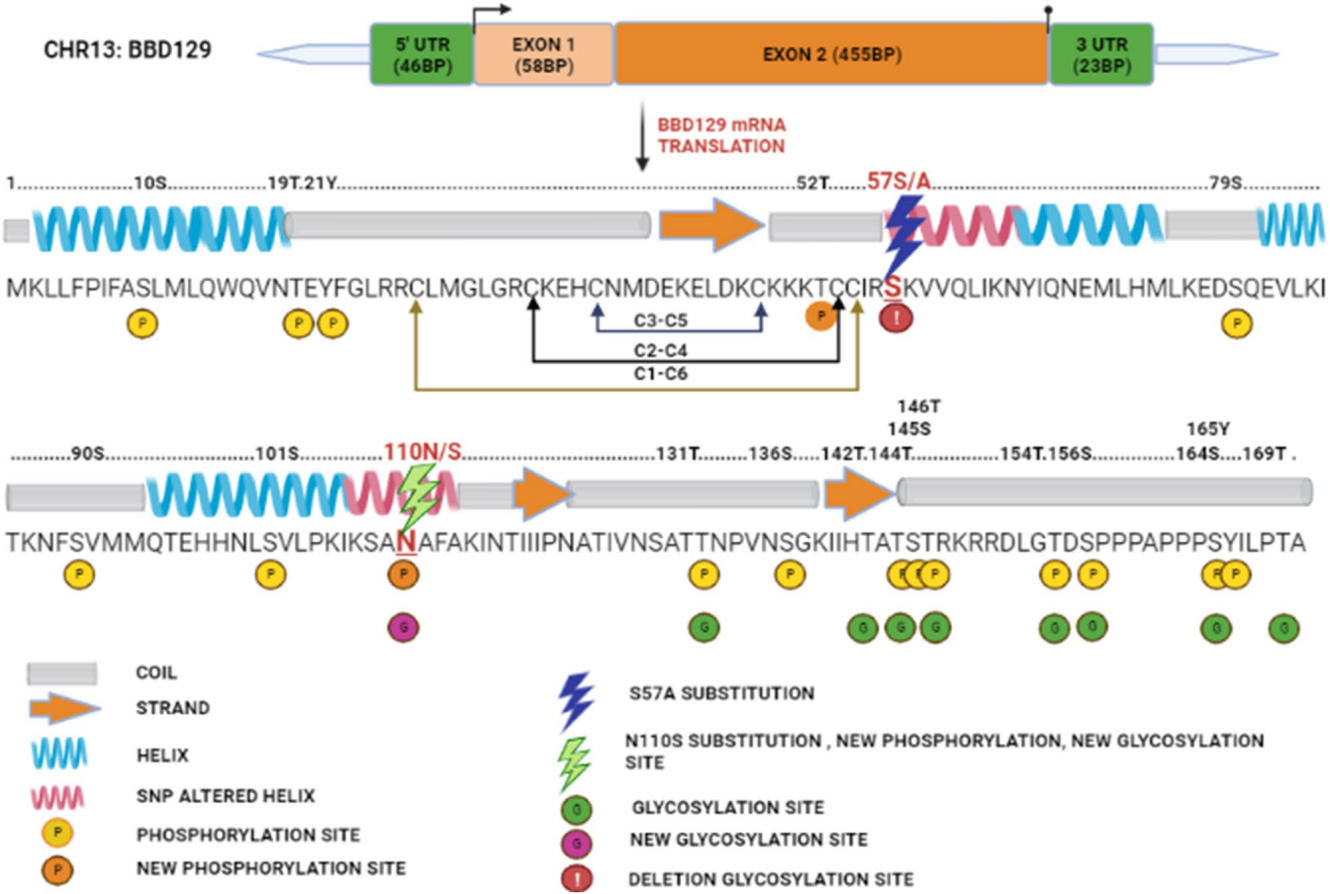
The overall pictorial representation of bioinformatic analyses of amino acid substitutions effects on secondary structure and post-translational modifications (PTMs) of *BBD129* protein. Dots represent the amino acid sequence positions, and mutated amino acid positions are shown in red alphabet on the dotted line. The amino acid substitutions have shown impact on helix secondary structure regions of *BBD129* protein and also, they were influencing the PTMs (viz. new threonine and serine phosphorylation sites and in/del O-glycosylation sites). The legends for secondary structure and PTMs are given in the figure.

**Figure 6:**
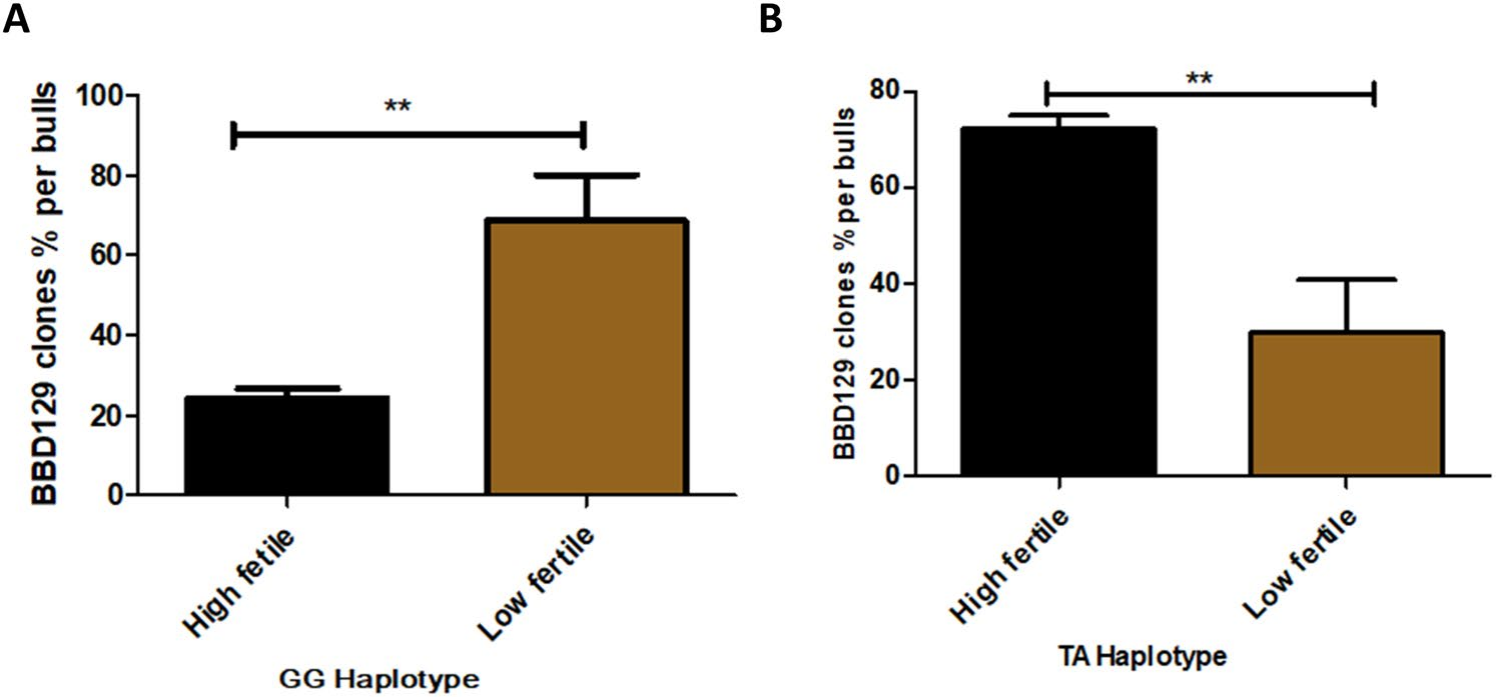
The *BBD129*-TA haplotype and *BBD129*-GG haplotype distribution in distinct fertility cross-bred bulls. The sequencing results have found that the cross-bred bulls produced a heterogeneous spermatozoa population bearing different haplotypes of the *BBD129* gene. (**A**) *BBD129*-TA haplotype was significantly more distributed in the high fertile bulls. (**B**) *BBD129*-GG haplotype was significantly more distributed in the low fertile bulls.

The *BBD129* gene was amplified from the sperm genomic DNA of distinct-fertility *Bos indicus x Bos taurus* bulls. The first exon of the *BBD129* gene has a band of 500 bp (Supplementary Fig. 6 A), and the second exon has a band of 600 bp (Supplementary Fig. 6 B,C). A total of 254 clones were sequenced to determine the distribution of *BBD129* polymorphisms in the genomic DNA of 11 distinct fertility bulls. There was no polymorphism found in the first exon of *the BBD129* gene. However, in exon second of the *Bos indicus x Bos taurus* cattle *BBD129* gene, we found two missense or non-synonymous SNPs at positions 169th (169 T > G, rs378737321) and 329th (329A > G, rs383285978) in the gDNA of both groups of fertility bulls (Figs. 4A; 5). The substitutions were also observed at the translated amino acid level of exon two of the *BBD129* gene. In the mutated *BBD129* protein, the serine amino acid at the 57th position was replaced by an alanine amino acid (S57A/srs378737321), while the asparagine amino acid at the 110th position was replaced by serine amino acid (N110S/rs383285978) (Figs. 4B; 5). Based on the types of polymorphisms and their positions, the *BBD129* gene was categorized into four haplotypes: *BBD129*-TA haplotype (169 T & 329A, when there was no polymorphism), *BBD129*-GA haplotype (169 T > G polymorphism, when polymorphism was found only at 169th position), *BBD129*-TG haplotype (329A > G polymorphism, when polymorphism was found only at 329^th^ position), and *BBD129*-GG haplotype (169 T > G & 329A > G, when polymorphisms were found at both places in the same amplified product of *BBD129* gene). The haplotype frequencies percentage distributions of *BBD129* gene in the HF bulls were (n = 105 clones): TA-haplotype (71.42%), GA-haplotype (1.90%), TG-haplotype (2.8%), and GG-haplotype (24.76%), while in case of LF group bulls, the haplotype frequencies percentage distributions of *BBD129* gene were (n = 149 clones): TA-haplotype (36.24%), GA-haplotype (0%), TG-haplotype (2.68%), and GG-haplotype (61.07%) (Fig. 6; Table 1). Two haplotypes, viz., *BBD129*-TA haplotype and *BBD129-*GG haplotype, were majorly distributed in both groups of bulls. There were very few single mutated clones, viz., GA-haplotype and TG-haplotype. We have found that the *BBD129* TA-haplotype was significantly more distributed in the HF bulls than in the LF cattle group (*P* = 0.0039). In contrast, *BBD129* GG-haplotype was significantly more distributed in the LF bulls than HF bulls (*P* = 0.0036). The sequencing results have found that the distribution of *BBD129* TA-haplotype (no mutation) was associated with a bull's high conception rate. In contrast, the double mutated *BBD129* GG-haplotype was associated with a bull's low conception rate (Table 1). The other two rare *BBD129* haplotypes (viz. *BBD129*-GA and *BBD129-*TG) were not analyzed further.

### The possible impacts of nsSNPs on the BBD129 protein post-translation modification

The glycosylation servers have predicted a deletion of the O-glycosylation site at the 57th amino acid position (0.46831 potential to be glycosylated, near to 0.5 threshold), and this alteration occurred due to the substitution of serine to alanine amino acid. Furthermore, one insertion of a new O-glycosylation site was found at the 110th amino acid position in the double mutated *BBD129* protein, and this alteration occurred due to the substitution of asparagine for serine amino acid (Figs. 4 and 5). The nsSNPs have shown no effect on the N-glycosylation of *BBD129* protein. The NetPhos 3.1 server found fifteen sites for phosphorylations in the non-mutated *BBD129* TA-haplotype protein, while two possible new phosphorylation sites were found in the double mutated *BBD129* GG-haplotype protein. The motif CKKK***T***CCIR (52nd amino acid) was a potential candidate for threonine phosphorylation with 0.545/0.503 scores for *Phosphokinase-G* and *Phosphokinase-A* enzymes. Another motif, IKSA***S***AFAK (110th amino acid), was found as a new potential candidate for serine phosphorylation for the *PKC* enzyme with a 0.617 score. These new phosphorylation sites have resulted from S57A and N110S amino acid substitutions, respectively (Fig. 5).

### The possible impact of nsSNPs on the BBD129 mRNA structure

**Figure 7:**
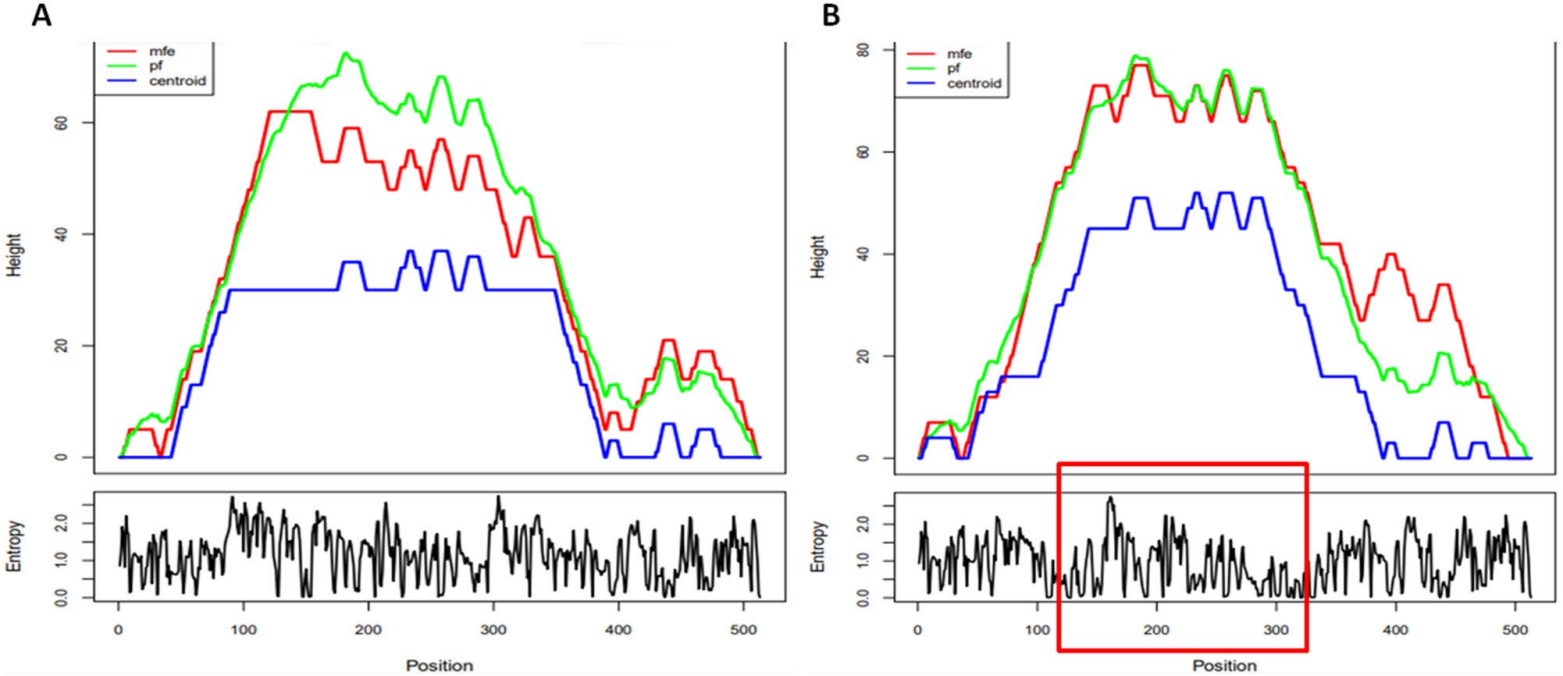
The impact of nsSNPs on the mRNA secondary structure properties was predicted by the RNAfold server. (**A**) RNAfold generated Mountain plot (minimum free energy, Partition function folding and centroid structure) curves for non-mutated *BBD129*-TA haplotype mRNA. (**B**) RNAfold generated a Mountain plot of double mutated *BBD129*-GG haplotype mRNA. The *BBD129* nsSNPs impacted the minimum free energy of mRNA secondary structure by influencing base-pairing properties and entropy.

**Figure 8:**
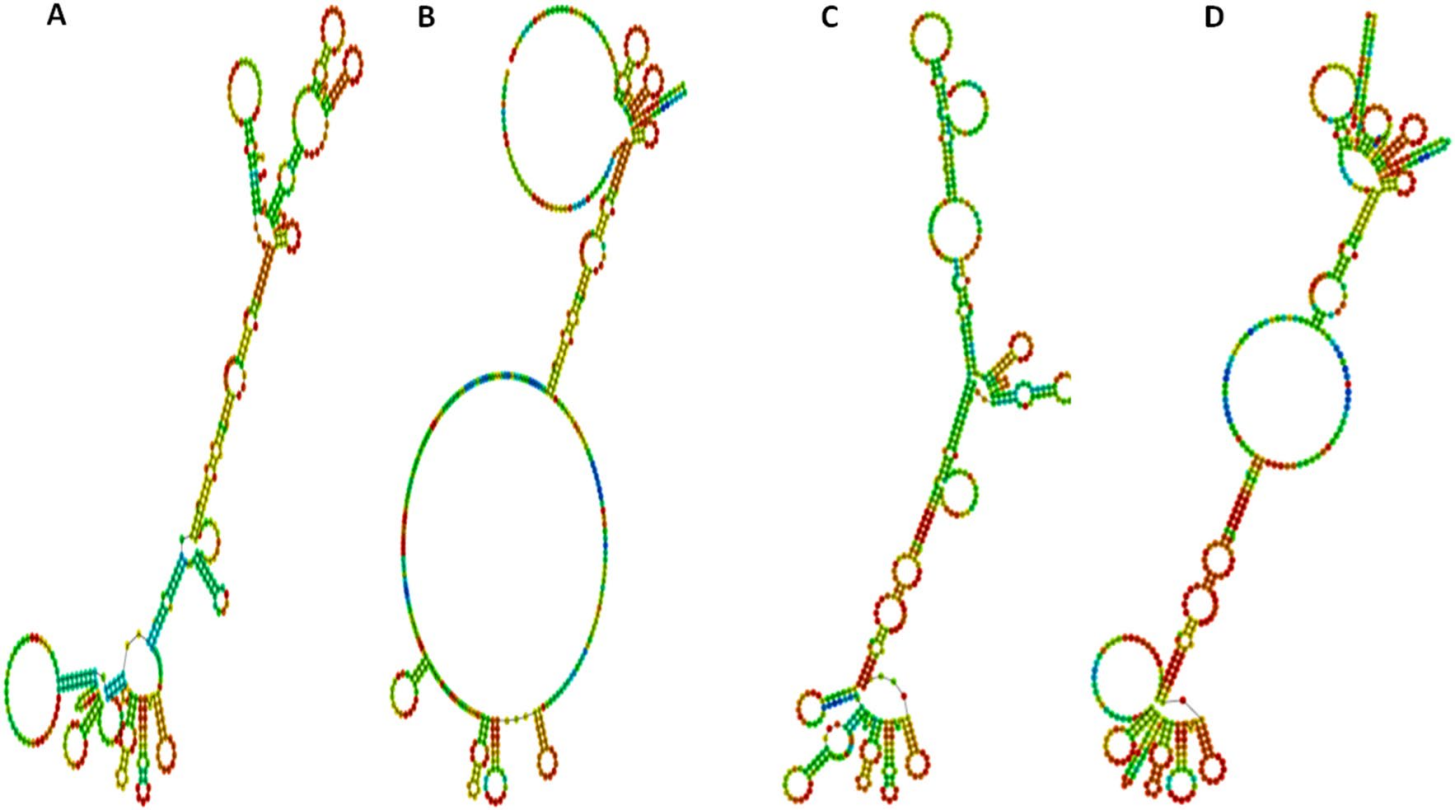
The prediction of *BBD129* mRNA secondary structure variations using RNAfold server. (**A**) The *BBD129*-TA mRNA secondary structure is based on the minimum free energy. (**B**) Centroid-free secondary structure of *BBD129*-TA mRNA. (**C**) The *BBD129*-GG mRNA secondary structure is based on minimum free energy. (**D**) Centroid-free secondary structure of *BBD129*-GG haplotype mRNA.

The nsSNPs of *BBD129* impacted the mRNA's minimum free energy (mfe) secondary structure by influencing base-pairing and entropy (Figs. 7, 8). The optimal mfe of non-mutated *BBD129*-TA haplotype mRNA was predicted at about − 93.77 kcal/mol, while in the case of double mutated *BBD129*-GG haplotype mRNA, the mfe was decreased to − 98.20 kcal/mol. The non-mutated *BBD129* thermodynamic ensemble's free energy was − 102.28 kcal/mol, and the ensemble diversity value was 142.05. In contrast, in the double mutated *BBD129* thermodynamic ensemble, free energy was decreased to − 107.88 kcal/mol, and the ensemble diversity value was 119.47 kcal/mol. The mfe of the native *BBD129* centroid secondary structure was about 46.40 kcal/mol, while in the case of double mutated *BBD129*, the mfe value increased to 61.78 kcal/mol. The changes in the MFE structural curve, the thermodynamic ensemble of RNA structural curve, the centroid structural curve and entropy changes in the double mutated *BBD129* mRNA can be seen in the mountain plot (Figs. 7, 8).

### The possible impacts of nsSNPs on the BBD129 protein stability

In *Polyphen-I*, the nsSNPs were benign in both the HumDIv and HumVar models of analysis. The deleterious effective score of the observed S57A substitution was 0.002 with 0.99 sensitivity and 0.30 specificity, while *Polyphen-I* predicted the harmful effective score of the N110S substitution as 0.004 with 0.97 sensitivity and 0.59 specificity (Supplementary Fig. 7). Similarly, *SIFT*, *SNAP*, and *MAPP* servers have found the first amino acid substitution S57A as deleterious, disease-causing and more structural distorter, while the second amino acid substitution, viz. N110S was predicted as a neutral substitution. The I-Mutant 2.0 server has predicted that both nsSNPs were structure distorters. Similarly, *Polyphen-I, PROVEN, SNPs & GO PhD-SNP*, *Meta-SNP*, and *PredictSNP* servers have found nsSNPs to have neutral and non-deleterious effects on the *BBD129* protein. The impacts of observed nsSNPs on the BBD129 protein are summarized in Table 2.

**Table 2:** Most advanced bioinformatics servers found that amino acid substitutions in *BBD129* protein lead to a detrimental effect by decreasing the protein stability and increasing disease-causing ability.

Tools	Mutation S57A (rs378737321) effect	Mutation N110S (rs383285978)effect
*PhD-SNP*	Neutral 0.118	Neutral 0.031
*SIFT*	Disease 0.030	Neutral 1.000
*SNAP*	Disease 0.515	Neutral 0.305
*Meta-SNP*	Neutral 0.163	Neutral 0.037
*I-Mutant2.0*	Stability decrease	Stability decrease
*SNPs & GO*	Neutral 0.118	Neutral 0.031
*PredictSNP*	Neutral 66%	Neutral 83%
*MAPP*	Disease 66%	Neutral 85%
*Polyphen-I*	Neutral 67%	Neutral 67%
*Polyphen-2*	Neutral 63%	Neutral 72%
*PROVEN*	Neutral -7.75	Neutral -0.024

### The possible impacts of nsSNPs on physiochemical properties of the BBD129 protein

The comparative *ProtParam* analysis of native *BBD129* and double mutated protein has shown significant alterations in the following properties: molecular weight, polar/non-polar amino acids, instability index, aliphatic index, and Grand Average of Hydropathy (GRAVY) (supplementary table 1). *The PSI-PRED* and *SOPMA* servers have predicted alterations in the *BBD129* protein secondary structure, especially in the helix and coils (Fig. 6). The SOPMA server predicted that the non-mutated *BBD129* protein sequence would have 33.77% alpha-helix, 1.32% beta-turn, 10.60% extended strand, and 54.30% random coil; however, in the double mutated *BBD129* protein, the alpha-helix was increased by 5.30%, and the beta-turn and random coil regions were decreased to about 1.32% and 3.97%, respectively. The *MUpro* server has predicted that both the amino acid substitutions were negatively affecting the protein stability of the *BBD129* protein. The substitution of serine to alanine amino acid at the 57th position (S57A_rs378737321) has decreased the protein stability by a confidence score of − 0.83258649 and − 0.9985692797182 in Support Vector Machine (SVM) and Neural Network Machine (NNM), respectively. The substitution of asparagine to serine amino acid at the 110th position (N110S_rs378737321) has decreased the protein stability by confidence scores of − 0.63001616 and − 0.65131013529855 in SVM and NNM, respectively (supplementary table 1).

### The possible impacts of nsSNPs on the BBD129 protein biological functions

As we hypothesized before that nsSNPs would reduce the *BBD129* protein's stability and influence its secondary structure and post-translational modifications, we used the Argot 2 server to estimate the potential effects of observed nsSNPs on the BBD129 protein's biological function. The analysis revealed that double-mutated *BBD129* protein had lower activities for all projected biological functions than non-mutated *BBD129* protein, indicating that the nsSNPs adversely impacted the biological functioning of the *BBD129* protein (Table 3). The overall results of all bioinformatics tools have been provided in Table 4.

**Table 3:** The list of biological functions comparison between *BBD129*-TA haplotype and mutated *BBD129*-GG haplotype proteins. The analysis of possible impacts of amino acid substitutions on *BBD129* protein were predicted by Argot 2.5 server.

GO ID	Name	Information content	*BBD129*-TA haplotype score	Mutated *BBD129*-GG haplotype score	Negative difference
GO:0,005,576	Extracellular region	6.5520748	5664.02	5617.75	− 46.27
GO:0,009,986	Cell surface	9.67	3005.11	2963.85	− 41.26
GO:0,006,952	Defense response	5.707	2774.83	2750.72	− 24.11
GO:0,045,087	Innate immune response	7.54	2242.19	2216.76	− 25.43
GO:0,042,742	Defense response to bacterium	11.843	3521.8	3481.86	− 39.94

**Table 4:** The overall conclusions of various bioinformatics tools used for the analyses of impact of polymorphisms on *BBD129* protein.

Bioinformatics tools	What it predicts	Our observation on *BBD129* substitutions	Biological logic by which it supports our hypothesis
MUpro tool	Prediction of Protein Stability Changes for Single-Site Mutations from Sequence	Decrease in *BBD129* protein stability & ∆∆G energy	Disturbance in functional structure formation (less availability, reduced antibacterial activity)
NetPhos tool	Predicts serine, threonine or tyrosine phosphorylation sites	Increase and frame shift in phosphorylation in *BBD129* protein	Early induction of acrosome and capacitation reactions i.e. Phagocytosis invitation
NetOGlyc 4	O-glycosylation predictions	Deletion and insertion of O-glyc sites in the *BBD129* protein	Alteration in glycosylation Pattern (problem in cumulus penetration—infertility)
Argot2	Function prediction (gene ontology)	*BBD129* substitutions lead to the decrease in biological functions	Decrease in their desired functions (non-functioning or reduced antibacterial activity)
PSI-PRED	Predicts secondary structure, disorder, and phi/psi dihedral angles of amino acids	*BBD129* substitutions lead to the changes in protein secondary structure, disorder	Disturbance in formation of functional protein structure (mis-folding less availability)
ProtParam	Instability index, thermostabilty, no. aa, MW, PI, charge, atoms, hydrophobicity, % charged amino acid, ext. coefficient	*BBD129* substitutions lead to the decrease in the atoms, molecular weight, protein Instability, thermostabilty, Aliphatic index, GRAVY (hydrophobicity)	Influence the protein functionality and antibacterial activity
O-Gly GlycoEP	O-linked glycosylation	Frame shift	Alteration in glycosylation Pattern (problem in cumulus penetration—infertility)
NetNGlyc	N-linked glycosylation	No change	No change
SOPMA	Protein secondary structure prediction	*BBD129* substitutions lead to the change in % of alpha helix, beta turn, extended strand, and random coil	Affect protein structure

## Discussion

Sperm in the testes can't fertilize an egg because they do not have enough maturity^73^. The complete maturation of testicular sperm occurs in the epididymal environment, where they are exposed to a variety of molecules, including highly negatively charged cysteine-rich glycosylated beta-defensin peptides secreted by epididymal epithelial cells and the coating of these glycoproteins, or exogenously addition of epididymal proteins (e.g., BDs), improves the sperm fertilization potential^74–76^. The prokaryotically produced bovine recombinant-*BBD126* protein enhances sperm motility and the capacity of sperm to traverse cervical mucus, but it does not enhance fertilizing ability^77,78^. This inability to increase sperm oocyte fertilization capacity might be attributed to the prokaryotic expression of BDs, since prokaryotic hosts lack post-translational modification machinery, and CA-BDs are well-known for their post-translational modifications (particularly glycosylations)^23,74^. The incompletion of their coding sequences or the availability of anticipated sequences in public databases may further restrict their usage in reproduction^79^. Therefore, it is necessary to properly characterize the genetic architecture of BDs to employ them to enhance the reproductive or fertility performance of bovines and other animals^34^. To address the insufficient characterization of defensins in bovines, we have defined one of the most important gene, *BBD129*, incross-bred cattle^23^. The bovine *BBD129* gene is an ortholog of the primate *DEFB126* gene^23^. RLM-RACE has been utilized to amplify matured capped and tailed mRNAs^80,81^, correctly detecting the 5' capped and 3' polyadenylation ends of mRNA^43,44^. The *Bos taurus* *BBD129* gene consists of two exons and 50 nucleotide UTRs at both ends of the mRNA^82^. We amplified the 5' and 3' ends of the *BBD129* mRNA with varied lengths of UTRs, and its coding region has a highly conserved sequence compared to the *DEFB129/BBD129* gene of other mammalian species. Its position on chromosome 13 was determined, preserving all the fundamental properties of beta-defensins as previously described^14–16^.This study's multiple sequence alignment and phylogenetic analysis showed a strong BBD129 amino acid sequence conservation association between the Bovidae and other mammalian families. The multiple sequence alignment of the *DEFB129/BBD129* gene's amino acid sequence with other mammalian species revealed structural changes in the exon second a protein-coding region while retaining disulfide bonds.

The expression pattern of *BBD129* mRNA in the MRT of cross-bred cattle demonstrates increased expression in the corpus-epididymis area with a region-specific expression pattern, similar to what has been described in the MRT of other mammalian species^18,23,30^. The sperm surface successively picks up BDs as they mature in the epididymis^14,18,29,83,84^. Unique expression patterns of *BBD129* in the mid-segment of the MRT epididymis indicate its presence as an intermediary and secretarial protein coat to be transported to the sperm surface. In a previous study in our lab on buffalo beta-defensin 129 (*BuBD129*), we demonstrated the region-specific expression of *BuBD129* in healthy MRT in *Bubalus bubalis*, defining its pleiotropic activities in addition to its traditional antimicrobial significance. The current finding confirms those findings^23,82^. The ortholog of bovine *BBD129*, primate *DEFB129*, is expected to play a crucial physiological function in sperm maturation via membrane modulations, motility, and immuno-protection in the female reproductive tract^14,27,85,86^. The human epididymal *DEFB129* protein binds to the sperm surface chemokine receptor *CCR6* and regulates calcium ion influx, inducing hypermotility in sperm. Exogenous additions of various recombinant-BDs to deficient spermatozoa considerably enhance their motility, viability, and antibacterial activity^37,78,87^. The fact that the cross-bred cattle-yak epididymal transcriptomics investigation verified a relationship between *BBD129* gene down-expression and male infertility demonstrates the significance of *BBD129* in male fertility^88,89^. Intense expression of the cross-bred cattle *BBD129* gene in healthy matured MRT tissues suggests the region-specific quantity of *BBD129* protein destined to be sequentially picked up by the sperm surface during epididymis maturation.

The non-synonymous mutations in the bovine neutrophil beta-defensin 4 (*BNBD4*) and *DEFB103* genes were reported with increased milk, fat, protein, lactose, dry matter, somatic cell counts, and mastitis disease resistance^75,78,90–92^. The polymorphisms in the BD genes can result in their selected environment expressions in the reproductive organs, indicating roles beyond typical antibacterial action^82,93,94^. The human *DEFB126* gene is highly polymorphic (77 SNPs), and two rs140685149 and rs11467497 polymorphisms are related to infertility by alerting sperm surface glycans, sperm motility, increased round somatic cells in semen, and sperm capacity to penetrate cervical mucus^10,11,33,35,77^. Twelve non-synonymous SNPs were found for the BBD129 gene in the bovine genome, however, their relationship to reproduction were not investigated^82^. In this work, we discovered two conserved non-synonymous SNPs (T169G and A329G) in the Indian cross-bred cow (*Bos taurus x Bos indicus)* genome, indicating the conservation nature of snSNPs in the bovine genome in various geographical regions of the globe. The *BBD129* gene sequences maintain thymine at the 169^th^ nucleotide position (alanine at the 57th amino acid position) and adenine at the 329^th^ nucleotide position (serine at the 110th amino acid position) in Indian bovine species. Sequencing indicated that Indian cross-bred bulls produced a diverse population of spermatozoa bearing four haplotypes of the *BBD129* gene. In the Indian cross-bred *Bos indicus x Bos taurus* cow genome, two haplotypes, *BBD129*-TA and double site mutated *BBD129*-GG, were predominantly prevalent. Others with single-site mutations in *BBD129* haplotypes were uncommon. The *BBD129* TA-haplotype was predominant in the bulls with high conception rates. In contrast, clone sequencing revealed that most of the heterogeneous sperm population was identified in the low fertile bulls with doubly mutant *BBD129* GG-haplotype, and their prevalence in the group of low fertile bulls may serve as a marker for bovine fertility.

Capacitation and acrosome reactions are connected with enhanced serine, tyrosine, and threonine phosphorylations and kinase activity^95–98^. Epididymal secretions and seminal plasma proteins are influential de-capacitation factors that preserve premature sperm capacitation and sperm acrosomal integrity before egg contact^99–101^. In this work, S57A and N110S amino acid substitutions in GG-*BBD129 protein* were expected to promote threonine and serine amino acid phosphorylations, which may stimulate sperm hyperactive motility early capacitation, and early acrosomal response or attract immune cells^102,103^.

It is generally known that epididymal BDs create a thick glycocalyx on the surface of sperm, which facilitates sperm functioning. The number of glycans on spermatozoa from different fertility buffalo bulls influences sperm-neutrophil phagocytosis and sperm-NETosis (sperm trapping)^104^. We found that the S57A amino acid substitution in the *BBD129* protein leads to the deletion of the O-glycosylation site (0.46831 potential) because the hydroxyl group carrying serine amino acid was replaced by non-hydroxyl alanine amino acid. In contrast, the N110S amino acid substitution has created space for a new site of O-glycosylation by converting asparagine amino acid to serine amino acid. These glycosylate variations may impact the sperm surface glycocalyx and the sperm's capacity for fertilization. All bioinformatic projection studies imply that snSNPs might significantly alter the function of the *BBD129* protein on sperm^11,22,35^.

Bioinformatic analyses underpin the possible causes of nsSNPs responsible for altered structure and functions of *BBD129* protein. The genomic variations in the *BNBD4* and Transmembrane Protein 95 (*TMEM95)* genes introduce a premature stop codon that leads to mRNA decay, and an altered codon resides within the transmembrane domain of *TMEM95*, most likely resulting in a disturbed anchorage of the truncated protein on the sperm plasma membrane, which affects sperm physiology^105,106^. The mountain curve plot analysis of *BBD129* nsSNP suggested that polymorphism influences the native mRNA structure and its base-pairing properties within the mRNA secondary structure. The polymorphisms in *BBD129* mRNA were destroying the native *BBD129* mRNA secondary structure and making it more complex by altering the base pairing characteristics, which could affect the transcription or translation processes. They may influence the bioavailability of *BBD129* protein in the epididymis or on the sperm surface.

In humans, Q47R amino acid substitution in the *PATE1* protein causes protein structural damage, leading to less bio-availability to the sperm membrane and reducing sperm motility associated with phosphorylations^107–109^. Bovine *SPAG11* gene polymorphisms were associated with sperm volume, sperm concentrations, and sperm motility^110^. In our study, the bioinformatics tools and *I-Mutant 2.0* servers suggested that both the substitutions resulted in protein structural distortion and negatively impacted the *BBD129* protein stability. The bioinformatics servers *ProtParam, Argot 2, MUpro, SOPMA,* and *PSI-PRED* analyses suggested that observed nsSNPs were disrupting the protein stability and altering protein physiochemical properties of the *BBD129*, leading to compromised biological functions or bio-availability. The substitutions of amino acids S57A and N110S in the *BBD129* protein found possible damaging effects on the hydrophobicity, net charge, aliphatic index content, molecular weight, and protein instability. However, bioinformatic servers' *SIFT, SNAP, SNPs & GO,* and *MAPP* analyses revealed that S57A amino acid substitution of BBD129 protein as a disease-causing substitution and N110S amino acid substitution as a neutral substitution, suggesting a possible reason behind the less conservation of single mutated *BBD129* haplotypes in the Indian cross-bred cattle genome. While other bioinformatic servers such as *Polyphen, PROVEN, PredictSNP,* and *PhD-SNP* analyses revealed that when both the nsSNP were present together (*BBD129*-TA and *BBD129-*GG haplotype), they were non-deleterious and thus, it could be a reason for still persistent double mutated *BBD129* haplotypes in the Indian cattle genome. Altogether, in-silico analyses suggest that polymorphisms in the *BBD129* protein negatively affected the protein conformation and protein stability, leading to the protein's less or non-functional.

## Conclusion

The current research found a link between *BBD129* gene polymorphisms and cross-bred cattle bull reproductive performance. The complete coding sequence of the *BBD129* mRNA in Indian cross-bred cattle demonstrated the conservation of protein function throughout bovine species. The higher expression of *the BBD129* gene in the corpus epididymis area may be essential for its absorption on the sperm surface as an intermediate sperm coat protein and provides insight into the *BBD129* coating dynamics on the sperm membrane in the MRT. The detailed analyses of polymorphism have shown that non-synonymous variants in the *BBD129* gene are convincingly associated with the reproductive status of Indian cross-bred bulls. In addition, the functional properties of the *BBD129* protein vary due to SNPs, which indicate the changed structural and conformational topology of the BBD129 mRNA and protein. These alterations in the *BBD129* protein might be responsible for the various capabilities of sperm to fertilize an egg. In-vitro validation assays for determining the altered fertilization of sperm owing to SNPs in *BBD129* of Indian cross-bred cattle need more research incorporating many validation populations.

## Supplementary Information

Supplementary Information 1.

Supplementary Information 2.

Supplementary Information 3.

## Data Availability

All the data provided in the main manuscript and supplementary files.
