# Supplementary material for: Analysis of amplification and association polymorphisms in the bovine beta-defensin 129 (BBD129) gene revealed its function in bull fertility
Source: Sci Rep. 2022 Nov 9;12:19042. doi: 10.1038/s41598-022-23654-3 (PMC9646896; doi:10.1038/s41598-022-23654-3)
Supplement: Supplementary file 1 — Supplementary Information 1. [file 41598_2022_23654_MOESM1_ESM.pdf]

Analysis of Amplification and Association Polymorphisms in the Bovine Beta-Defensin 129 (*BBD129*) Gene revealed its Function in Bull Fertility.

Subhash Solanki, Poonam Kashyap, Syed Azmal Ali, Ashutosh vats, Vijay Kumar, Martina Pukhrambam, Rakesh Kumar, Sachinandan De, and Tirtha Kumar Datta*

Supplementary file 1

The DEFB129 or BBD129 amino acid sequences used for the Phylogenetic tree construction

>RACE_BBD129

MKLLFPIFASLMLQWQVNTEYFGLRRCLMGLGRCKEHCNMDEKELDKCKKKTCCIRSK
VVQLIKNYIQNEMLHMLKEDSQEVLKITKNFSVMMQTEHHNLSVLPKIKSANAFAKINT
IIIPNATIVNSATTNPVNSGKIIHTATSTRKRRDLGTDSPPPAPPPSYILPTA

>Mus_musculus defensin beta 23 (Defb23), mRNA

MKLLFPLFASLMLQSQVKSEFATVKRCLVGFGKCKDSCLADETQMQHCKAKKCCIGPK
VSELIKSYLRQEIPHIPDEDIVEMMRTKKNSTEEMQGKQALMALFQSRAAKNLLSNTNS
AMGPNAFPVKSVTTRTRRRRMGATASTKMHIKQSKDSANAAPRPRPGPP

>Bubalus_bubalis defensin beta 129 (DEFB129), mRNA

MKLLFPIFASLMLQWQVNTEYFGLRRCVMGLGRCKEYCNMDEKELDKCKKKKCCIRS
KVVQLIKNYIQNEMLHMLKEDSQEVLKITKNFSFMMQTEHHNLSVLPKIKSASAFANIN
TIIIPNATIVNSATTNPVNSGKIIHTATSTKKRRDSGTDSPPPAPPPSYTPPTA

>Bos_indicus_x_Bos_taurus defensin beta 129 (DEFB129), mRNA

MKLLFPIFASLMLQWQVNTEYFGLRRCLMGLGRCKEHCNMDEKELDKCKKKTCCIRA
KVVQLIKNYIQNEMLHMLKEDSQEVLKITKNFSVMMQTEHHNLSVLPKIKSASAFAKIN
TIIIPNATIVNSATTNPVNSGKIIHTATSTRKRRDLGTDSPPPAPPPSYILPTA

>Bos_mutus defensin, beta 129 (DEFB129), mRNA

MKLLFPIFASLMLQWQVNTEYFGLRRCVMGLGRCKEHCNMDEKELDKCKKKTCCIRA
KVVQLIKNYIQNEMLHMLKEDSQEVLKITKNFSVMMQTEHHNLSVLPKIKSASAFAKIN
TIIIPNATIVNSATTNPVNSGKIIHTATSTRKRRDLGTDSPPPAPPPSYILPTA

>Bos_taurus defensin beta 129 (DEFB129), mRNA

MKLLFPIFASLMLQWQVNTEYFGLRRCLMGLGRCKEHCNMDEKELDKCKKKTCCIRSK
VVQLIKNYIQNEMLHMLKEDSQEVLKITKNFSVMMQTEHHNLSVLPKIKSANAFAKINT
IIIPNATIVNSATTNPVNSGKIIHTATSTRKRRDLGTDSPPPAPPPSYILPTA

>Bos_indicus defensin beta 129 (DEFB129), mRNA

MKLLFPIFASLMLQWQVNTEYFGLRRCLMGLGRCKEHCNMDEKELDKCKKKTCCIRA
KVVQLIKNYIQNEMLHMLKEDSQEVLKITKNFSVMMQTEHHNLSVLPKIKSASAFAKIN
TIIIPNATIVNSATTNPVNSGKIIHTATSTRKRRDLGTDSPPPAPPPSYILPTA

>Camelus_ferus defensin beta 129 (DEFB129), mRNA

MKLLFPIFASLMLQYQVNTEYFGLGRCVMGFGRCKDHCAMDEKEVEKCRKKKCCIGP
KVVQLIKSYLQNEMLHGLEEDSQKLLKTTKNSSLMMQTKYHIFSLLPKIKSSNPFANINTI
IIPNGTAGDSATANPMISRKTTHTSTSTKSDTKERRDSATDSLPLAPPP

>Camelus_bactrianus defensin, beta 129 (DEFB129), mRNA

MKLLFPIFASLMLQYQVNTEYFGLGRCVMGFGRCKDHCAMDEKEVEKCRKKKCCIGP
KVVQLIKSYLQNEMLHGLEEDSQKLLKTTKNSSVMMQTKYHIFSLLPKIKSSNPFANINT
IIIPNGTAGDSATANPMISRKTTHTSTSTKSDTKERRDSATDSLPLAPPP

>Camelus_dromedarius defensin beta 129 (DEFB129), mRNA

MKLLFPIFASLMLQYQVNTEYFGLGRCVMGFGRCKDHCAMDEKEVEKCRKKKCCIGP
KVVQLVKSYLQNEMLHGLEEDSQKLLKTTKNSSVMMQTKYHIFSLLPKIKSSNPFANIN
TIIIPNGTAGDSATANPMISRKTTHTSTSTKSDTKERRDSATDSLPLAPPP

>Capra_hircus defensin beta 129 (DEFB129), mRNA

MKLLFPIFASLMLQWQVNTEYFGLRRCVMGLGRCKEHCNMDEKELDKCKKKKCCIRS
KVVQLIKNYIQNEMLHMLKEDSQEVLKITKNVSVMMQTKHHNLSALPKIKSANAFANV
NTIIIPNATIVNSATTNPVNSGKIIHTATPTKKRRDSGTDSPPPAPPPSYTLPTA

>Felis_catus defensin beta 129 (DEFB129), mRNA

MKLLFPIFASLMLQYQVNTEYFGLRRCLMGFGRCKDYCAVGETEIQKCKKRKCCIGQK
VVQMIKNYMQNEMSHTLEGNSQEHLQVTKNSDALIQTKYQILSLLPRTKSISPFANVHP
LLIPNATTVNSAITNPTTSCKITYTAISAKNNTTESRDSASDSSPPAPPP

>Bison_bison bison defensin, beta 129 (DEFB129), mRNA

MKLLFPIFASLMLQWQVNTEYFGLRRCVMGLGRCKEHCNMDEKELDKCKKKTCCIRA
KVVQLIKNYIQNEMLHMLKEDSQEVLKITKNFSVMMQTEHHNLSVLPKIKSASAFAKIN
TIIIPNATIVNSATTNPVNSGKIIHTATSTRKRRDLGTDSPPPAPPPSYILPTA

>Ovis_aries defensin beta 129 (DEFB129), mRNA

MKLLFPVFASLMLQWQVNTEYFGLRRCVMGLGRCKEHCNMDEKELDKCKKKKCCIRS
KVVQLIKNYIQNEMLHVLKEDSQEVLKITKNVSVMMQTKHHNLSVLPKIKSANAFANIN
TIIIPNATIVNSATSNPVNSGKIIHTATSTKKRRDSGTDSLPPAPPPSYTLPTA

>Equus_caballus defensin beta 129 (DEFB129), mRNA

MKLLFPIFASLVLQYQVNTEYFGLRRCLMGLGRCKDHCATNEREIQQCKNKRCCIGPKL
VQVIKSYMKNELSHIFEENSQQLLKTTKSSSVVMQTKHHILSSLSKIKSTGSFAKINTITIP
SAIPVNSTTINPKITGKITYTATSTKSDTKESKDSATDSPPTAPPP

> Homo sapiens defensin beta 129 (DEFB129), mRNA

MKLLFPIFASLMLQYQVNTEFIGLRRCLMGLGRCRDHCNVDEKEIQKCKMKKCCVGPK
VVKLIKNYLQYGTPNVLNEDVQEMLKPAKNSSAVIQRKHILSVLPQIKSTSFFANTNFVII
PNATPMNSATISTMTPGQITYTATSTKSNTKESRDSATASPPPAPPPPNILPTPSLELEEAE
EQ

>Canis_familiaris beta-defensin 129 (CBD129) mRNA, complete cds

MKLLFTIFASLMLQYQVNTEYFGLRRCLMGFGRCRDHCAMAEKEIQKCKKKKCCIGPK
VVQMIKNYMQNEMSHTLEGSQEQLPINKNFDVEMQTKNRILSLLPKSKSISPFANVSTLII
SNTTNINSVIANPVFSGKTSHTAISTKSDTKERRDSDTDSPPPAPPP

>Canis_lupusfamiliaris defensin beta 129 (DEFB129), mRNA

MKLLFTIFASLMLQYQVNTEYFGLRRCLMGFGRCRDHCAMAEKEIQKCKKKKCCIGPK
VVQMIKNYMQNEMSHTLEGSQEQLPINKNFDVEMQTKNRILSLLPKSKSISPFANVSTLII
SNTTNINSVIANPVFSGKTSHTAISTKSDTKERRDSDTDSPPPAPPP

>Macaca_fascicularis defensin beta 129 (DEFB129), mRNA

MKLLFPIFASLMLQYQVNTEFIGLRSCLMGFGRCRDHCNVDEKEIQKCKMKKCCVGPK
VVKLIKNYLQYGTPNVLNEDVQEMLKPAENSSAVIQRKHILSILPQIKSINFFANTNLVIIP
NATPVNSATVSTMTSGQITYTATSTKSNTKESRDSATASPPPAPPPPNILPTPSLELEEAEE
Q

>Mus_pahari defensin beta 129 (Defb129), mRNA

MKLLFPLFASLMLQSQVKSEFASMKRCLMGFGKCKDRCLAEETQMQNCKAKKCCVGP
KVTELIKSYLRQEIPHIPDEDVVEMMKMEKNSTEEMQGKQALMAVFQSRAAKYLLSNT
NSAMGPNAFPVKSVTTRIRRRHMGVTASTETHIKQSRDSANAAPQPRPGPP

>Mus_caroli defensin beta 129 (Defb129), mRNA

MKLLFPLFASLVLQSQVKSEFVAMKRCIMGFGKCKDSCLAEETQMQHCKAKKCCIGPK
VTELIKSYLRQEIPHIPDDDIVEMMRIEKNSSEEMQGKQALMALSQSRAAKNLLSNANSA
VGPNAFPVRSVTTRTRRRRMGDTASTEMHIKQSRDSAKAAPQPRPGPP

>Macaca_nemestrina defensin beta 129 (DEFB129), mRNA

MKLLFPIFASLMLQYQVNTEFIGLRSCLMGFGRCRDHCNVDEKEIQKCKMKKCCVGPK
VVKLIKNYLQYGTPNVLNEDVQEMLKPAENSSAVIQRKHILSILPQIKSINFFANTNLVIIP
NATPVNSATVSTMTSGQITYTATSTKSNTKESRDSATASPPPAPPPPNILPTPSLELEEAEE
Q

>Pan_troglodytes defensin beta 129 (DEFB129), mRNA

MKLLFPIFASLMLQYQVNTEFIGLRRCLMGLGRCRDHCNVDEKEIQKCKMKKCCVGPK
VVKLIKNYLQYGTPNVLNEDVQEMLKPAKNSSAVIQRKHILSVLPQIKSTSFFANTNFVII
PNATPMNSATISTMTPGQITYTATSTKSNTKESRDSATASPPPAPPPPNILPTPSLELEEAE
EQ

>Sus_scrofa defensin beta 129 (DEFB129), mRNA

MKLLFPIFASLMLQYQVNTEYFGLGRCVMGLGRCKDHCAVNEKEIDKCKKKKCCIGPK
GIQLIKSYLQNEMIRTLEEVAKKNITKNSKVVTPSKYRPLSHLPEIKSTNPFASINTTIIPNG
TTVNSTTTNSTTSRRSTHPATSTKSDTKKRRDSPTESPPADSSPIDPADSMT

>Gorilla_gorilla defb129 gene for beta defensin 129

MKLLFPIFASLMLQYQVNTEFIGLRRCLMGLGRCRDHCNVDEKEIQKCKMKKCCVGPK
VVKLIKNYLQYGTPNVLNEDVQEMLKPAKNSSAVIQRKHILSVLPQIKSTSFFANTNFVII
PNATPMNSATISTMTPGQITYTATSTKSNTKESRDSATASSPPAPPPPNILPTPSLELEEAE
EQ

> Ailuropoda_melanoleuca defensin beta 129 (DEFB129), mRNA

MKLLFPIFASLLLQYQVNTEYFGLRRCLMGFGRCKDHCAVDEKEIQKCKKKKCCIGPK
MVQMITNYMQNEMSNTLGEGSQEHLQTTKNSDAEIQTKSQILSLLPQIKRMSPFAHSNT
LISPNATNVNSLLTNPVFSGNISYTAVATKRDTKESRNSAADSPQPAPPPQMLPTT

>Loxodonta_africana defensin beta 129 (DEFB129), mRNA

MKLLFPIFASLMLQSQVNTEFLGLKKCLMGFGKCKDHCTVDEKEIDKCKKKKCCIGPKV
VQIIKNFIQIEMLHAFEENSQGLLKNYNNSNAMIQKKNHVTSILPKIMSISPSTNTNTVIIT
NTTTLNSIVTSTAASTKSDTTESRDSPIVSIPPTPPP

>Equus_asinus defensin beta 129 (DEFB129), mRNA

MKLLFPIFASLVLQYQVNTEYFGLRRCLMGLGRCKDHCATNEREIQQCKNQRCCIGPKL
VQVIKSYVKNELSHIFEENSQQLLKTTKSSSVVMQTKHHILSSLSKIKSTGSFAKINTITIPS
AIPVNSTTINPKITGKITYTATSTKSDTKESKDSATDSPPTAPPPQRLPVP

>Hylobates_moloch defensin beta 129 (DEFB129), mRNA

MKLLFPIFASLMLQYKVNTEFIGLRRCLMGFGRCRDHCNVDEKEIQKCKMKKCCVGPK
VVKLIKNYLQYGTPNVLNEDVQEMLKPAKNSSAVIQRKHILSVLPQIKSTSFFANTNFVII
PNATPMNSATISTVTPGQITYTAASTKSNIKESRDSATASPPPAPPPPNTLPTP

Supplementary file 2

Note: Nucleotide sequences given below in underlined sequences were matched with cross-bred predicted BBD129 gene and non-underlined sequences were found as untranslated region (UTR) of BBD129 mRNA.

FASTA*Bos taurus x Bos indicus* (Cross-bred cattle) RLM-RACE BBD129 5’ Inner PCR

5’CCCAGATCCAAGGCTTCTTCTCTTGGAAGAAGAGAAGCTTCCAGCCATGAAGCTC
CTTTTCCCTATCTTTGCCAGCCTTATGCTACAGTGGCAGGTGAACACAGAATACTTTG
GCTTGAGAAGATGCCTAA3’

FASTA*Bos taurus x Bos indicus* (Cross-bred cattle) RACE BBD129 3’ Inner PCR

5’TGTCATGATGCAGACCGAACATCATAATTTATCTGTTCTGCCCAAAATCAAAAGTG
CCAATGCTTTTGCCAAGATCAACACCATCATCATCCCAAATGCCACCATTGTGAACT
CTGCCACCACCAACCCCGTGAACTCAGGGAAGATAATACACACTGCTACTTCTACCA
GAAAAAGAAGAGATTTAGGCACTGACTCCCCACCACCAGCACCACCTCCATCGTAT
ATACTTCCGACAGCATAACTGGAGCTGGAGAAGCAGATGAG3’

FASTA*Bos taurus x Bos indicus* (Cross-bred cattle) BBD129 cDNA PCR

5’AGAGCACTGCAACATGGATGAAAAAGAGTTAGATAAATGCAAAAAGAAAACATG
TTGTATTAGATCAAAAGTGGTTCAACTGATAAAAAACTACATACAAAATGAAATGCT
CCATATGCTTAAAGAGGACTCTCAGGAAGTGCTAAAAATTACCAAGAATTTTAGTGT
CATGATGCAGACCGAACATCATAATTTATCTGTTCTGCCCAAAATCAAAAGTGCCAA
TGCTTTTGCCAAGATCAACACCATCATCATCCCAAATGCCACCATTGTGAACTCTGC
CACCACCAACCCCGTGAACTCAGGGAAGATAATACACACTGCTACTTCTACCAGAA
AAAGAAGAGATTTAGGCACTGACTCCC3’

FASTA*Bos taurus x Bos indicus* (Cross-bred cattle) BBD129 mRNA complete coding sequence

5’CCCAGATCCAAGGCTTCTTCTCTTGGAAGAAGAGAAGCTTCCAGCCATGAAGCTC
CTTTTCCCTATCTTTGCCAGCCTCATGCTACAGTGGCAGGTGAACACAGAATACTTTG
GCTTGAGAAGATGCCTAATGGGTTTGGGGAGATGCAAAGAGCACTGCAACATGGAT

GAAAAAGAGTTAGATAAATGCAAAAAGAAAACATGTTGTATTAGATCAAAAGTGGT
TCAACTGATAAAAAACTACATACAAAATGAAATGCTCCATATGCTTAAAGAGGACT
CTCAGGAAGTGCTAAAAATTACCAAGAATTTTAGTGTCATGATGCAGACCGAACATC
ATAATTTATCTGTTCTGCCCAAAATCAAAAGTGCCAATGCTTTTGCCAAGATCAACA
CCATCATCATCCCAAATGCCACCATTGTGAACTCTGCCACCACCAACCCCGTGAACT
CAGGGAAGATAATACACACTGCTACTTCTACCAGAAAAAGAAGAGATTTAGGCACT
GACTCCCCACCACCAGCACCACCTCCATCGTATATACTTCCGACAGCATAACTGGAG
CTGGAGAAGCAGATGAG3’

NCBI BankIt GenBank Submissions MW900256

Submission Number # 2449612

Supplementary Figure S1:

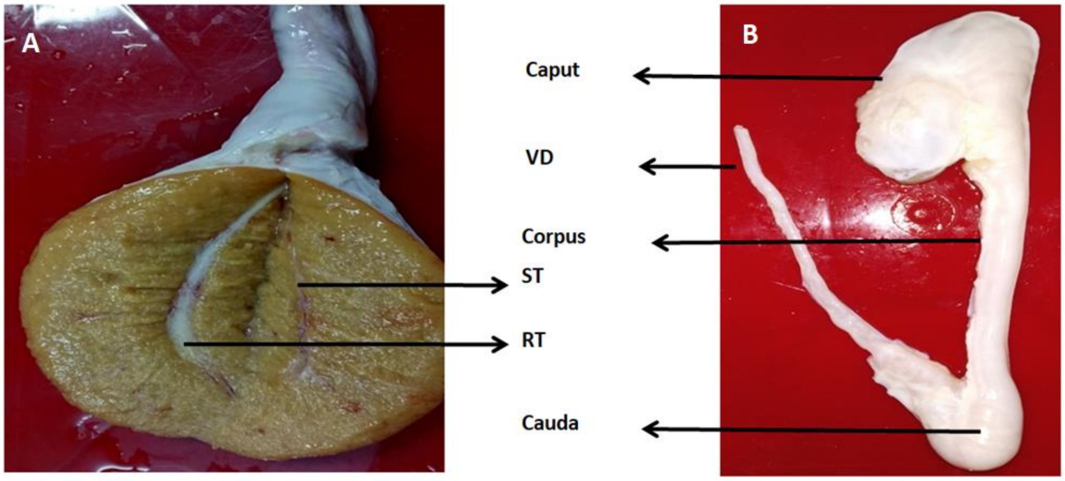

Cattle male reproductive organ. A) Matured testis showing rete testis (TS), seminiferous tubule (ST). B) Epididymis showing caput, corpus, cauda, and vas-deferens (VD).

Supplementary Figure S2

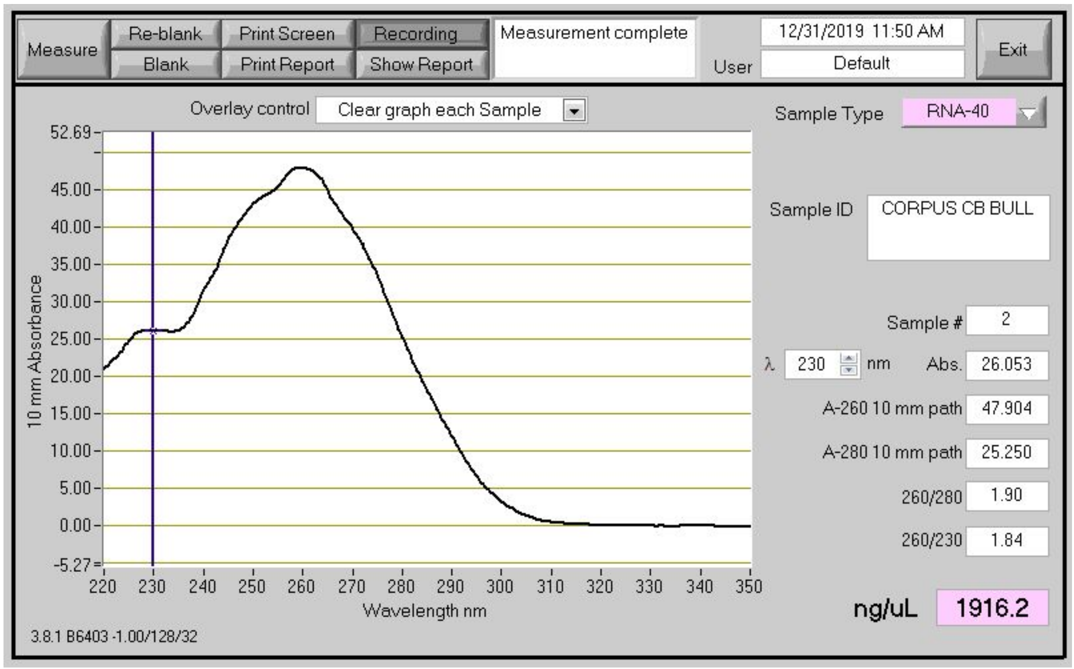

RNA quality: The extracted RNA was quantified by NanoDrop spectrophotometer.

Supplementary Figure S3

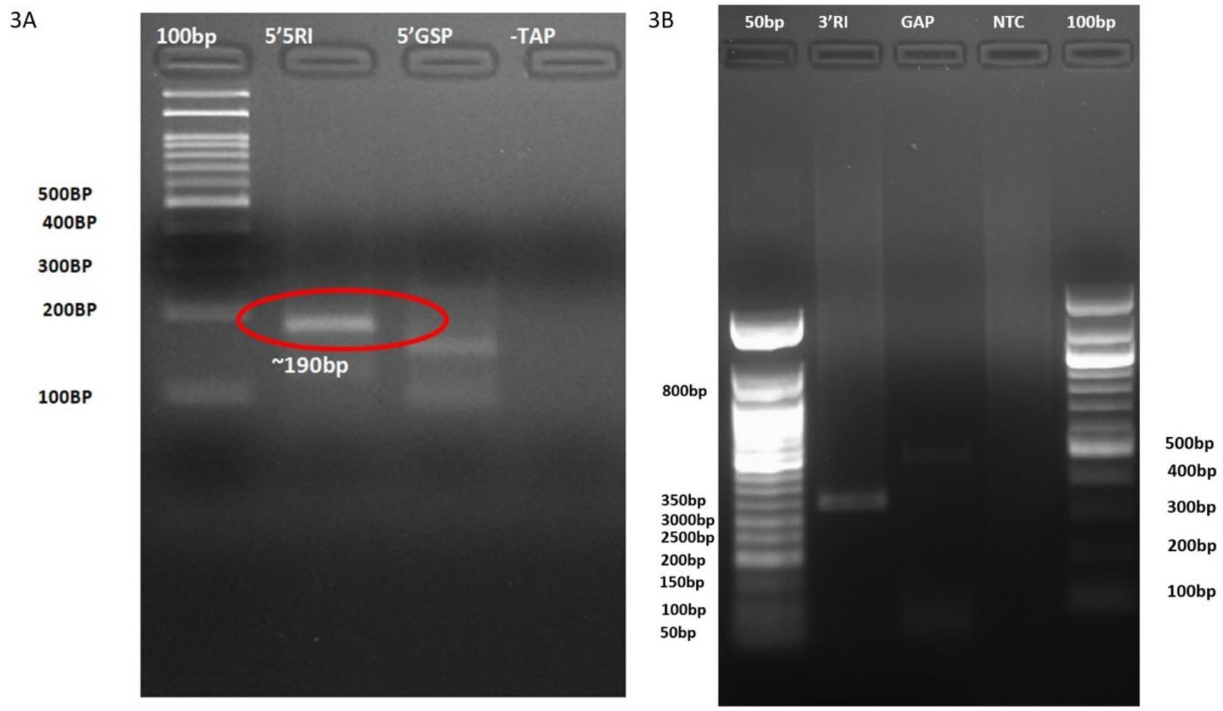

The 5' and the 3' end amplifications of cross-bred *BBD129* mRNA using RLM-RACE methodology. A) 5’ RLM-RACE PCR (5RI) has shown a band of ~190 bp. 5’ gene-specific PCR (5’GSP) was run as a positive control, and negative TAP was run to see amplification from degraded or incomplete BBD129 mRNA. B) The 3’ RACE PCR (3RI) has a band of ~300 bp. Glyceraldehyde 3-phosphate dehydrogenase (GAP) and Non template control (NTC) were run as positive and negative PCR control, respectively. 100bp = 100 base pair DNA ladder, 50bp = 50 base pair DNA ladder.

Supplementary Figure S4

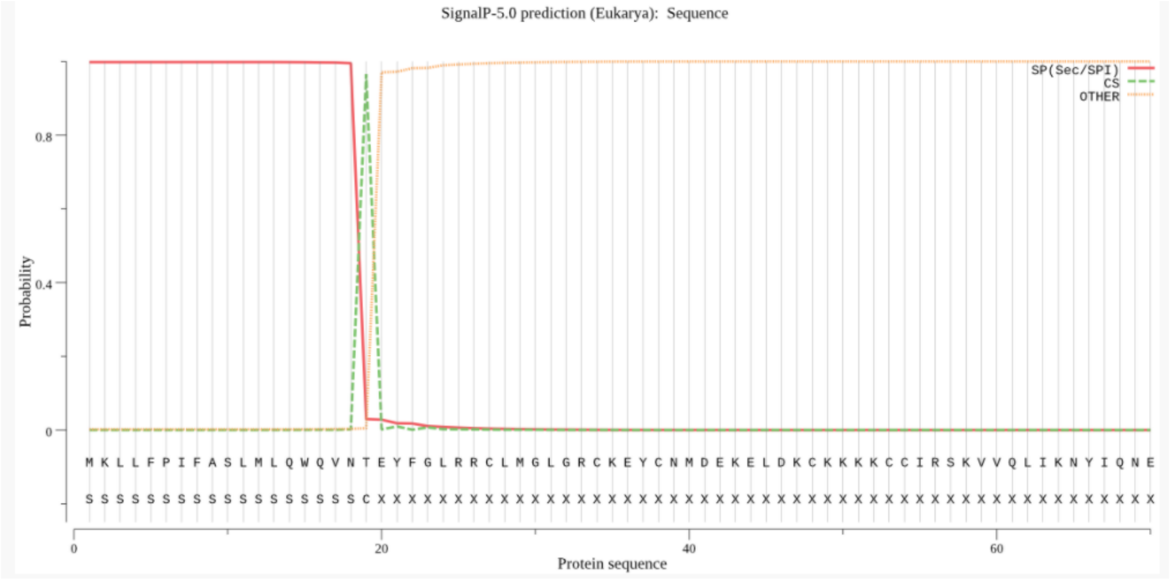

The signal peptide prediction in the RLM-RACE amplified *the Signal-IP 5.0 server did BBD129 gene*. The *BBD129* gene has a signal peptide of 19 amino acids long, and it bears a protease site between the 19th and 20th amino acids.

Supplementary Figure S5

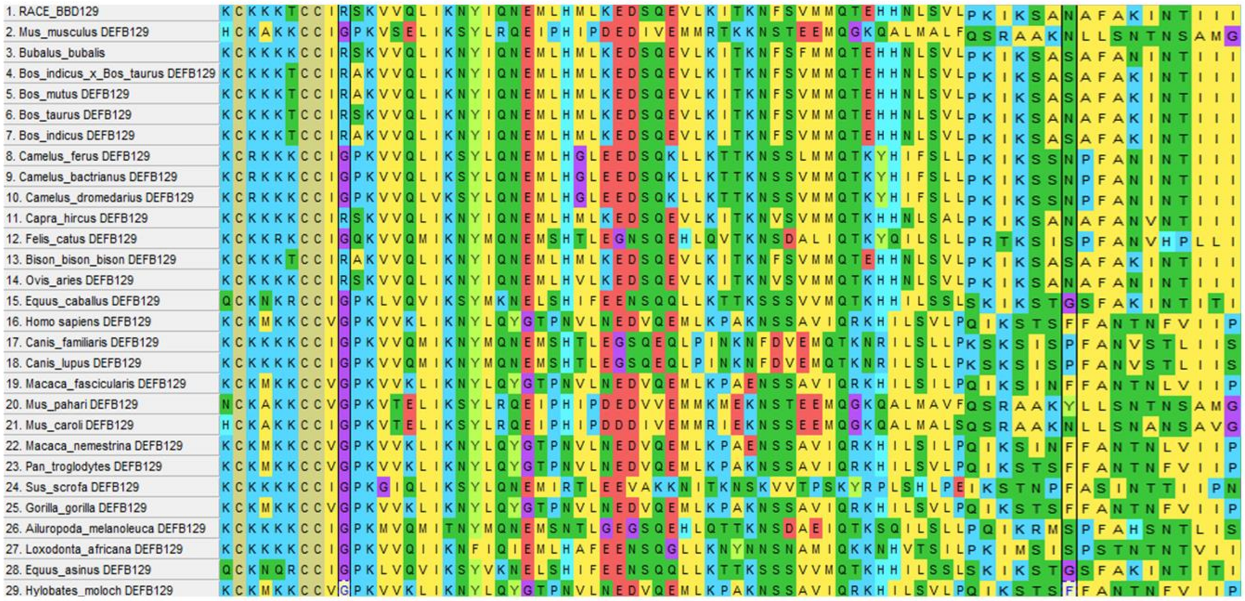

Multiple sequence alignment (MSA) of *BBD129* amino acid sequences of different mammalian species extracted from NCBI. MSA has found *BBD129* protein structural variations. RLM-RACE amplified BBD129

gene of Cross-bred cattle (*Bos indicus x Bos taurus*) has shown some amino acid sequence variations in relation to other species.

Supplementary Figure S6

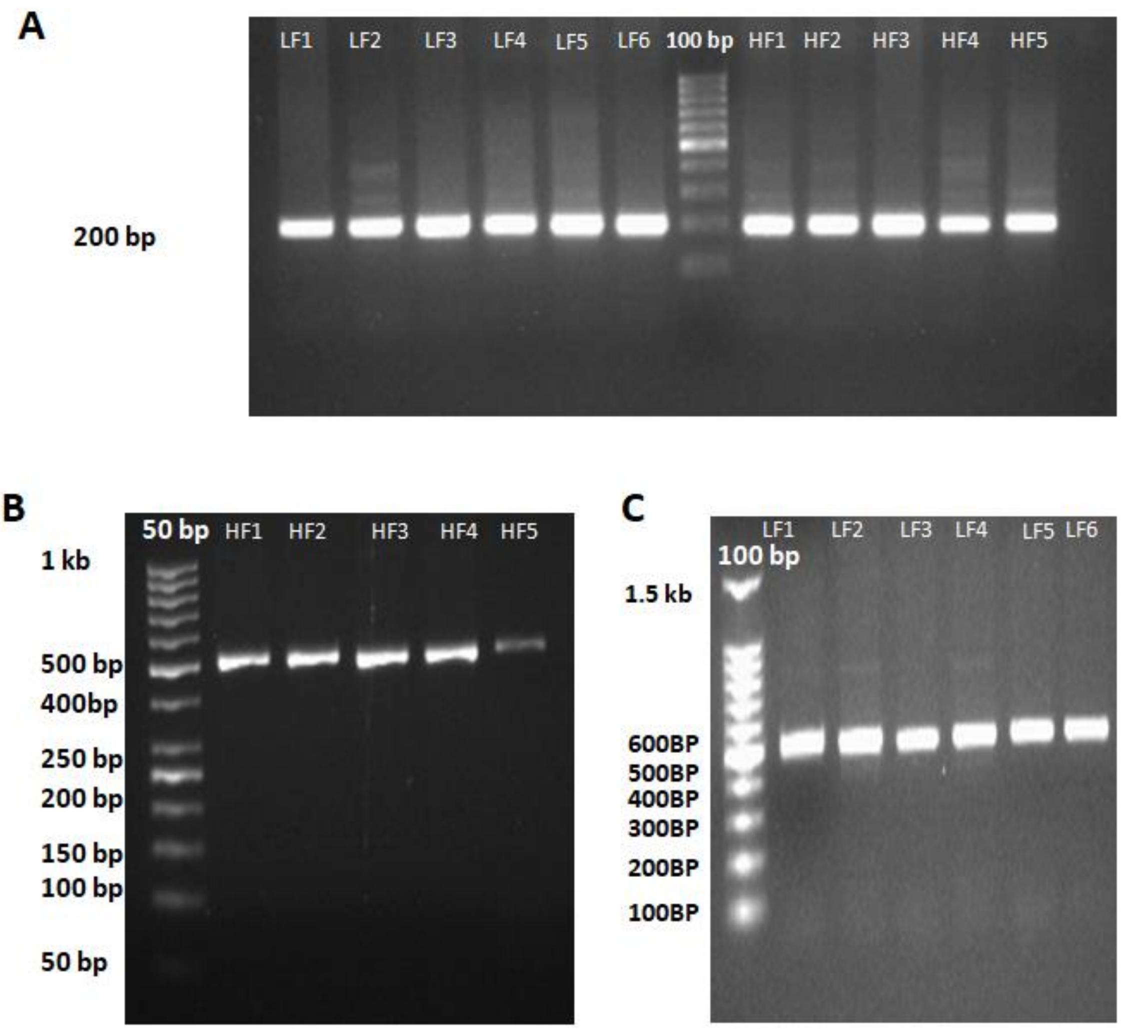

The genomic DNA amplification of *Bos indicus x Bos taurusBBD129* gene. A) The genomic DNA PCR amplification of *BBD129* exon-1 from distinct fertility cross-bred bulls. B The genomic DNA PCR amplification of *BBD129* exon-2 has shown a band around 500bp in the high fertile cross-bred bulls. C) The genomic DNA PCR amplification of *BBD129* exon-2 has shown a band around 500bp in the low fertile cross-bred bulls Abbreviations: HF = High fertile cross-bred bull, LF = Low fertile cross-bred bull.

Supplementary Figure S7

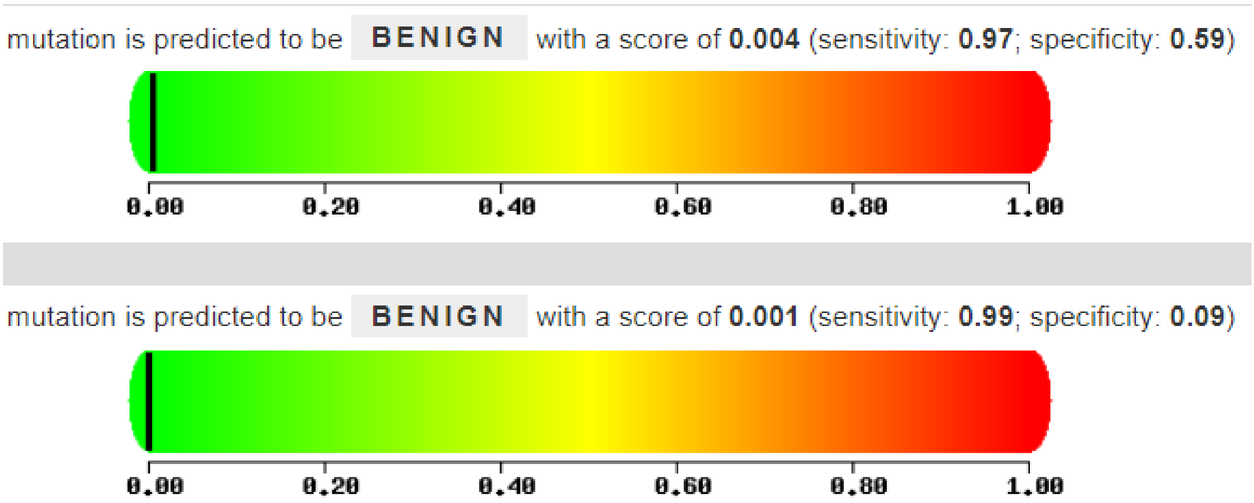

Polyphen prediction of the impact of nsSNPs on the BBD129 protein (HumDIv and HumVar models).

***This single file contains 9 supplementary tables mentioned in the manuscripts.**

TABLES:

Supplementary Table S1: The list of primers used to amplify the 5' and 3' ends, expressional analysis and genomic DNA of *Bos indicus x Bos taurusBBD129* gene.

S.No.	Primer Name	Length	Sequence (5’->3’)
1	5' RACE Outer Primer	24	GCTGATGGCGATGAATGAACACTG
2	5' RACE Inner Primer	28	CGCGGATCCGAACACTGCGTTTGCTGGC
3	5' RACE exon specific primer	21	AGAGCACTGCAACATGGATGA
4	5’ RACE gene specific outer primer	23	CTTCCTGAGAGTCCTCTTTAAGC
5	5’ RACE gene specific inner primer	20	ATTAGGCATCTTCTCAAGCC
6	3' RACE Outer Primer	23	GCGAGCACAGAATTAATACGACT
7	3' RACE Inner Primer	32	CGCGGATCCGAATTAATACGACTCACTATAGG
8	3’ RACE gene specific outer primer	21	AGAGTACTGCAACATGGATGA
9	3’ RACE gene specific inner primer	23	GCTTAAAGAGGACTCTCAGGAAG
10	cDNA forward primer	26	AGAGCACTGCAACATGGATGAAAAAG
11	cDNA reverse primer	26	AAGAAGAGATTTAGGCACTGACTCCC
12	GAPDH forward primer	20	CACTCCCAACGTGTCTGTTG
13	GAPDH reverse primer	21	AGACAACCTGGTCCTCAGTGT
14	eE2 forward primer	20	ACATCATGATCGACCCCGTC
15	eE2 reverse primer	20	TGGCCACATACATCTCAGCA
16	Exon first forward primer	25	GAGGTCTTCCTTCTTGTTACACTGA
17	Exon first reverse primer	22	AACCCAGATCCAAGGCTTCTTC
18	Exon second forward primer	21	CCATCAACGTCCCTAACCACT
19	Exon second reverse primer	21	CTGTGAGGCTCCTGTGAGAAA

Supplementary Table S2: The classifications of Indian cattle bulls into two group of distinct fertility. The conception rate cutoff value for low fertile bulls was selected 32.00 % while for high fertile bulls the conception rate cutoff value was more than 50.00%.

	Cattle Bull No.	No. A.I. at 1st conception	Total No. of A.I.	CR at 1st & 2nd A.I.	Over All CR % (Total A.I.)
High fertile cattle Bulls	HF-1	103	134	53.40	50.75
	HF-2	66	78	56.06	55.13
	HF-3	70	86	57.14	54.65
	HF-4	89	189	51	57.30
	HF-5	62	128	62.90	57.81
Low fertile cattle Bulls	LF-1	81	105	30.86	29.52
	LF-2	76	116	31.58	30.17
	LF-3	45	67	24.44	28.36
	LF-4	77	114	25.97	27.19
	LF-5	84	173	30.95	31.79
	LF-6	64	94	31.25	30.85

Abbreviations: A.I= Artificial Insemination; CR=Conception rate

Supplementary Table S3: List of RT-qPCR comparative expressions of cattle BBD129 gene between different tissues of crossbred cattle (*Bos indicus x Bos taurus*) male reproductive system.

Tukey's Multiple Comparison Test	Mean Diff.	q	Significant? P < 0.05?	Summary
ST vs RT	-4.508	15.48	Yes	***
ST vs CAPUT	-4.087	14.03	Yes	***
ST vs CORPUS	-14.2	48.76	Yes	***
ST vs CAUDA	-10.59	36.34	Yes	***
ST vs VD	-6.07	20.84	Yes	***
RT vs CAPUT	0.4218	1.448	No	ns
RT vs CORPUS	-9.696	33.29	Yes	***
RT vs CAUDA	-6.077	20.86	Yes	***
RT vs VD	-1.561	5.36	Yes	*
CAPUT vs CORPUS	-10.12	34.73	Yes	***
CAPUT vs CAUDA	-6.499	22.31	Yes	***
CAPUT vs VD	-1.983	6.808	Yes	**
CORPUS vs CAUDA	3.62	12.43	Yes	***
CORPUS vs VD	8.135	27.93	Yes	***
CAUDA vs VD	4.516	15.5	Yes	***

Abbreviations: ST= Seminiferous Tubule; RT= Rete testis; VD= Vasa deferentia

Supplementary Table S4: List of different features for novel characterized *Bos indicus x Bos taurus* (Cross-bred cattle) BBD129 gene.

Features		Indian cattle RLM-RACE BBD129
Gene size (mRNA)		582 bp
Exon 1 size		58 bp
Exon 2 size		455 bp
Intron size		~1.66 kb
5' UTR size		46 bp
3'UTR size		23 bp
Chromosome location		13
Disulfide linkage		1-6, 2-4, 3-5
O-glycosylation sites		8
N-glycosylation sites		3
Phosphorylation sites		15
Secondary structure beta sheet		3
CCR6 chemokine receptor binding score		74.798
Lipopolysaccharide binding score		70.194
Protein binding score		26.022
Defense response score		1669.530
Innate immune response score		99.588
Extracellular region score		6114.130
Coding potential score		2.98311
Blast summary	Hit num	5
	Hit score	46.124993
	Frame score	709.171665683427
ORF_FrameFinder	Coverage	92.78%
	Log-odds score	90.76
	Type	Partial
ORF Information	Start	47(AUG)
	End	560 (TAA)
	Length	511
Uniprot Hit ID		Q30KS5
		Q9H1M3
		Q9H1M3
		Q32ZG9
		Q30KP0

Abbreviations: RLM= RNA ligase mediated Rapid amplification of cDNA ends, UTRs= Untranslated Regions; ORF= Open reading Frame

Supplementary Table S5: List of biological function prediction for the*Bos indicusx Bos taurus* (Cross-bred cattle) BBD129 gene by Argot 2 tool.

Function predicted	GO ID	Name	Indian cattle BBD129 score	Internal confidence	Information content
Molecular functions	GO:0031721	CCR6 chemokine receptor binding	74.798	0.317	15.120
	GO:0001530	Lipopolysaccharide binding	70.194	0.360	11.015
	GO:0005515	Protein binding	26.022	0.639	5.095
Biological process	GO:0006952	Defense response	1669.530	0.997	5.706
	GO:0045087	Innate immune response	99.588	0.285	7.540
Cellular component	GO:0005576	Extracellular region	6114.130	0.813	6.55

Supplementary Table S6: List of clone haplotypes observed in the cattle bulls of distinct fertility.

Cattle Bulls No.	Conception rate (%)	BBD129 clones	BBD129 TA Haplotype% (169T/329A)	BBD129 GG Haplotype % (169G/329G)	BBD129 GA Haplotype % (169G)	BBD129 TG Haplotype % (329G)	Remarks
HF-1	50.75%	18	72.22%	16.66%	0%	11.11%	Total clone sequenced = 105 TA haplotype = 72.21% GG haplotype = 23.16% GA haplotype = 2.2% TG haplotype = 2.2%
HF-2	55.13%	23	65.21%	34.78%	0%	0%	
HF-3	54.65%	17	82.35%	17.64%	0%	0%	
HF-4	57.30%	25	64%	24%	12%	0%	
HF-5	57.81%	22	77.27%	22.72%	0%	0%	
							Geo_mean was calculated to normalize sample side
LF-1	29.52%	22	13.63%	86.36%	0%	0%	Total clone sequenced= 149 TA haplotype = 29.18% GG haplotype = 68.65% TG haplotype = 2.68%
LF-2	30.17%	22	45.45%	45.45%	0%	9.09%	
LF-3	28.36%	21	38.09%	61.90%	0%	0%	
LF-4	27.19%	19	0%	100%	0%	0%	
LF-5	31.79%	19	10.52%	89.48%	0%	0%	
LF-6	30.85%	46	67.39%	28.26%	0%	4.34%	

Abbreviations: HF = High fertile, LF = Low fertile, CR = Conception rate, AI = Artificial insemination, T = Thymine, A = Adenine, G = Guanine.

Supplementary Table S7: ProtParam *in silico* predicted physiochemical parameters comparison between non-mutated BBD129 and double mutated BBD129 protein.

**Properties**	**BBD129 TA haplotype**	**BBD129 GG (rs378737321& rs383285978) Haplotype**
No. of amino acids	170	170
molecular weight	19255.71	19212.68 (-43.03) Da
Theoretical PI value	9.56	9.56
Negatively charged (Asp + Glu):	13	13
positively charged (Arg + Lys):	24	23
Carbon	850	849 (-1)
Hydrogen	1395	1394 (-1)
Nitrogen	237	236 (-1)
Oxygen	242	241 (-1)
Sulfur	14	14
Formul	$C_{850}H_{1395}N_{237}O_{242}S_{14}$	$C_{849}H_{1394}N_{236}O_{241}S_{14}$
Total number of atoms	2738	2734 (-4)
Ext. coefficient (M-1 cm-1, at 280 nm measured in water)	10345	10345
Abs 0.1% (=1 g/l), assuming all Cys residues are reduced	0.518	0.538 (+0.020)
Instability index	39.11	36.53 (-2.50)
Aliphatic index	87.76	88.35 (+0.59)
Grand average of hydropathicity (GRAVY)	-0.275	-0.244
Ala	5.30%	5.9% (+0.6)
Arg	4.10%	4.10%
Asn	7.10%	6.5% (-0.6)

Abbreviations: Ala=alanine; Arg=arginine, Asn=aspartame

Supplementary Table S8: Mupro bioinformatic prediction of single amino acid substitutions impact on the BBD129 protein.

**BBD129 SNP: S57A (rs378737321&)**	**BBD129 SNP: N110S (rs383285978)**
Original Amino Acid: Serine amino acid (38th)	Original Amino Acid: Asparagine amino acid (91st)
Substitute Amino Acid: Alanine amino acid	Substitute Amino Acid: Serine amino acid
Prediction Results: Prediction of the sign (direction) of energy change using SVM and neural network with a smaller sequence window	Prediction Results: Prediction of the sign (direction) of energy change using SVM and neural network with a smaller sequence window
Method 1: Support Vector Machine, use sequence information only.	Method 1: Support Vector Machine, use sequence information only.
Effect: DECREASE the stability of protein structure.	Effect: DECREASE the stability of protein structure.
Confidence Score: -0.83258649	Confidence Score: -0.63001616
Method 2: Neural Network, use sequence information only.	Method 2: Neural Network, use sequence information only.
Effect: DECREASE the stability of protein structure.	Effect: DECREASE the stability of protein structure.
Confidence Score: -0.998569279497182	Confidence Score: -0.65131013529855

Supplementary Table S9: List of comparison of O-glycosylations between non-mutated BBD129 and double mutated BBD129 proteins predicted by GlycoEP standard predictor server.

**Non-mutated BBD129TA haplotype**					**Double mutated BBD129 GG** (rs378737321& rs383285978) haplotype			
Position	Residue	Score	Prediction		Position	Residue	Score	Prediction
10	S	-1.57125	Non-glycosylated		10	S	-1.57125	Non-glycosylated
19	T	-1.67062	Non-glycosylated		19	T	-1.67062	Non-glycosylated
52	T	-1.92694	Non-glycosylated		52	T	-2.14787	Non-glycosylated
57	S	0.46831	Non-glycosylated		57			
79	S	-1.47706	Non-glycosylated		79	S	-1.47706	Non-glycosylated
86	T	-1.68423	Non-glycosylated		86	T	-1.68423	Non-glycosylated
90	S	-0.8051	Non-glycosylated		90	S	-0.8051	Non-glycosylated
95	T	-0.09626	Non-glycosylated		95	T	-0.09626	Non-glycosylated
101	S	-0.59928	Non-glycosylated		101	S	-0.52942	Non-glycosylated
108	S	-0.11774	Non-glycosylated		108	S	-0.0479	Non-glycosylated
110					110	S	0.406	Potential Glycosylated
117	T	0.830371	Potential Glycosylated		117	T	0.842429	Potential Glycosylated
124	T	0.438753	Potential Glycosylated		124	T	0.438753	Potential Glycosylated
128	S	0.392211	Potential Glycosylated		128	S	0.392211	Potential Glycosylated
130	T	0.392211	Potential Glycosylated		130	T	0.392211	Potential Glycosylated
131	T	0.660606	Potential Glycosylated		131	T	0.660606	Potential Glycosylated
136	S	1.195607	Potential Glycosylated		136	S	1.195607	Potential Glycosylated
142	T	-0.56668	Non-glycosylated		142	T	-0.56668	Non-glycosylated
144	T	-0.38312	Non-glycosylated		144	T	-0.38312	Non-glycosylated
145	S	-0.28422	Non-glycosylated		145	S	-0.28422	Non-glycosylated
146	T	0.096935	Potential Glycosylated		146	T	0.096935	Potential Glycosylated
154	T	1.147249	Potential Glycosylated		154	T	1.147249	Potential Glycosylated
156	S	0.554275	Potential Glycosylated		156	S	0.554275	Potential Glycosylated
164	S	0.76828	Potential Glycosylated		164	S	0.76828	Potential Glycosylated
169	T	0.268058	Potential Glycosylated		169	T	0.268058	Potential Glycosylated

Abbreviations: T=threonine, Y=Tyrosine, S=serine

Supplementary TableS10: List of comparison of phosphorylations sites between native BBD129 and double mutated BBD129 proteins predicted by NetPhos 3.1 server.

**BBD129_TA haplotype**						**BBD129 GG haplotype** (rs378737321& rs383285978)					
Site	a.a	Motif	Score	Enzyme	Comment	Site	a.a	Motif	Score	Enzyme	Comment
10	S	PIFASLMLQ	0.749	PKA	YES	10	S	PIFASLMLQ	0.749	PKA	YES
19	T	WQVNTEYFG	0.507	cdc2	YES	19	T	WQVNTEYFG	0.507	cdc2	YES
21	Y	VNTEYFGLR	0.511	EGFR	YES	21	Y	VNTEYFGLR	0.511	EGFR	YES
52	T	CKKKTCCIR				52	T	CKKKTCCIR	0.545	PKG	YES
52	T	CKKKTCCIR				52	T	CKKKTCCIR	0.503	PKA	YES
79	S	LKEDSQEVL	0.984	unsp	YES	79	S	LKEDSQEVL	0.984	unsp	YES
79	S	LKEDSQEVL	0.603	DNAPK	YES	79	S	LKEDSQEVL	0.603	DNAPK	YES
79	N	LKEDSQEVL	0.559	ATM	YES	79	N	LKEDSQEVL	0.559	ATM	YES
90	S	TKNFSVMMQ	0.934	unsp	YES	90	S	TKNFSVMMQ	0.934	unsp	YES
101	S	HHNLSVLPK	0.579	PKC	YES	101	S	HHNLSVLPK	0.579	PKC	YES
110	S	IKSASAFAK				110	S	IKSASAFAK	0.617	PKC	YES
131	T	NSATTNPVN	0.610	PKC	YES	131	T	NSATTNPVN	0.610	PKC	YES
136	S	NPVNSGKII	0.933	unsp	YES	136	S	NPVNSGKII	0.933	unsp	YES
144	T	IHTATSTRK	0.691	PKC	YES	144	T	IHTATSTRK	0.691	PKC	YES
145	S	HTATSTRKR	0.986	unsp	YES	145	S	HTATSTRKR	0.986	unsp	YES
145	T	HTATSTRKR	0.914	PKC	YES	145	T	HTATSTRKR	0.914	PKC	YES
145	S	HTATSTRKR	0.464	GSK3	YES	145	S	HTATSTRKR	0.464	GSK3	YES
146	T	TATSTRKRR	0.904	PKC	YES	146	T	TATSTRKRR	0.904	PKC	YES
146	T	TATSTRKRR	0.718	unsp	YES	146	T	TATSTRKRR	0.718	unsp	YES
154	T	RDLGTDSPP	0.738	PKC	YES	154	T	RDLGTDSPP	0.738	PKC	YES
156	S	LGTDSPPPA	0.840	unsp	YES	156	S	LGTDSPPPA	0.840	unsp	YES
156	S	LGTDSPPPA	0.591	cdk5	YES	156	S	LGTDSPPPA	0.591	cdk5	YES
164	S	APPPSYILP	0.568	PKA	YES	164	S	APPPSYILP	0.568	PKA	YES
164	S	APPPSYILP	0.504	GSK3	YES	164	S	APPPSYILP	0.504	GSK3	YES
165	Y	PPPSYILPT	0.708	unsp	YES	165	Y	PPPSYILPT	0.708	unsp	YES

Full images of agarose gel *Only for supplementary data (Main manuscript contain cropped images).

Figure 1

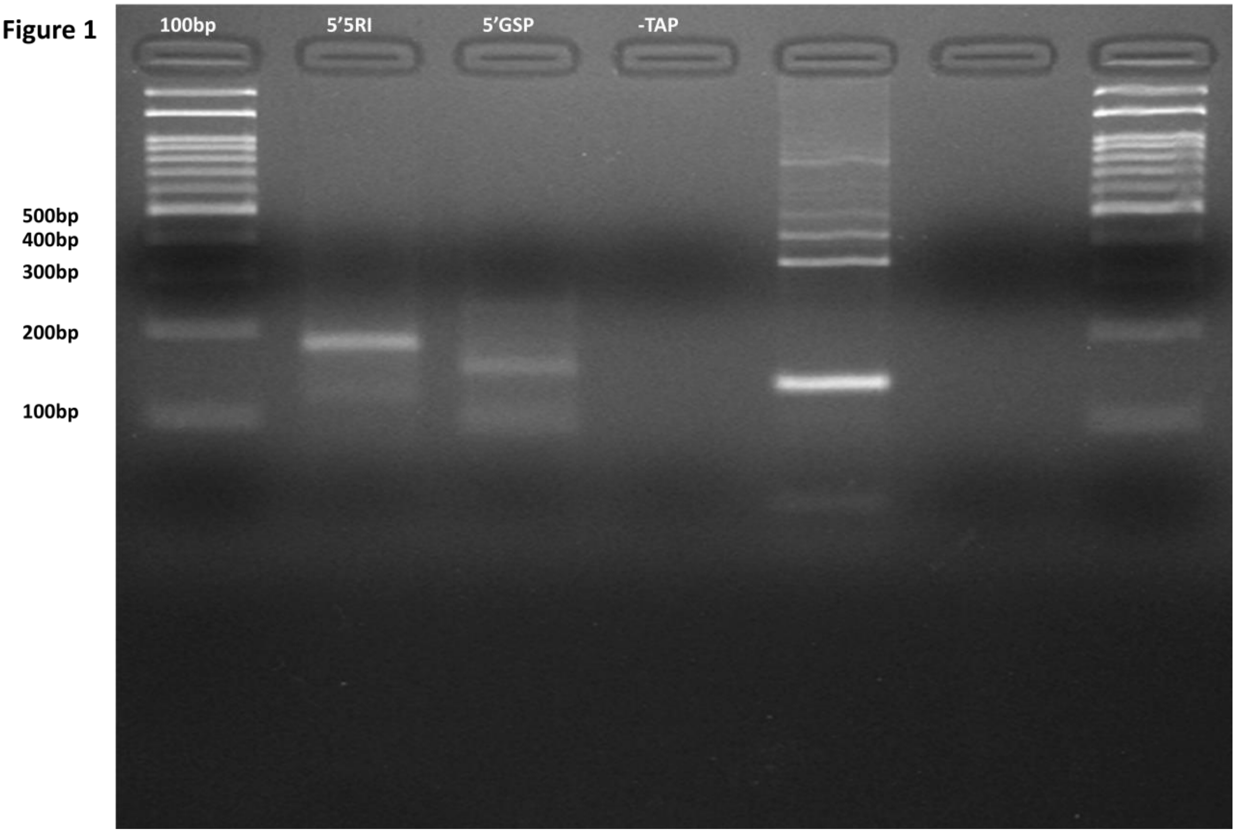

Supplementary Figure 3 (Full view): The 5' and the 3' end amplifications of crossbred *BBD129* mRNA using RLM-RACE methodology. A) 5' RLM-RACE PCR (5RI) has shown a band of ~190 bp. 5' gene-specific PCR (5'GSP) was run as a positive control, and negative TAP was run to see amplification from degraded or incomplete BBD129 mRNA. 100bp = 100 base pair DNA ladder, 50bp = 50 base pair DNA ladder.

* Lane numbers 5 to 7 are not of our interest, Lane5= non-specific PCR product, Lane6=empty, Lane7= 100bp DNA ladder.

Figure 1

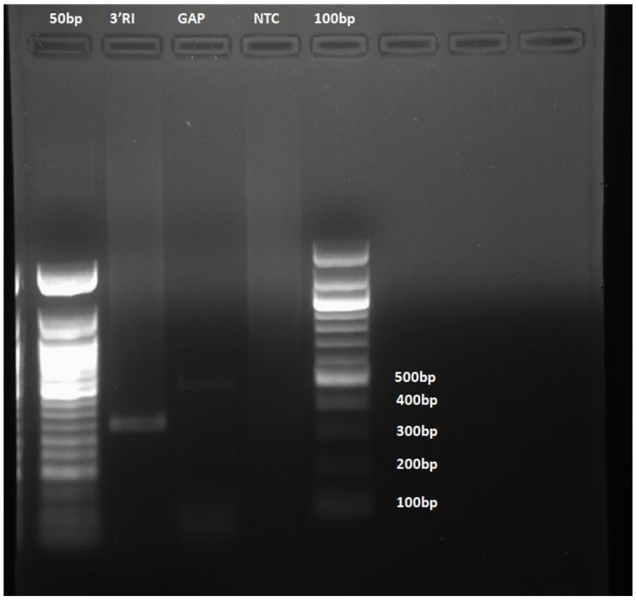

Supplementary figure 3 (full view): The 5' and the 3' end amplifications of crossbred *BBD129* mRNA using RLM-RACE methodology. 1B) The 3' RACE PCR (3RI) has a band of ~300 bp. Glyceraldehyde 3-phosphate dehydrogenase (GAP) and Non template control (NTC) were run as positive and negative PCR control, respectively. 100bp = 100 base pair DNA ladder, 50bp = 50 base pair DNA ladder.

*Lanes 6 to 8 is empty.

Figure 5A

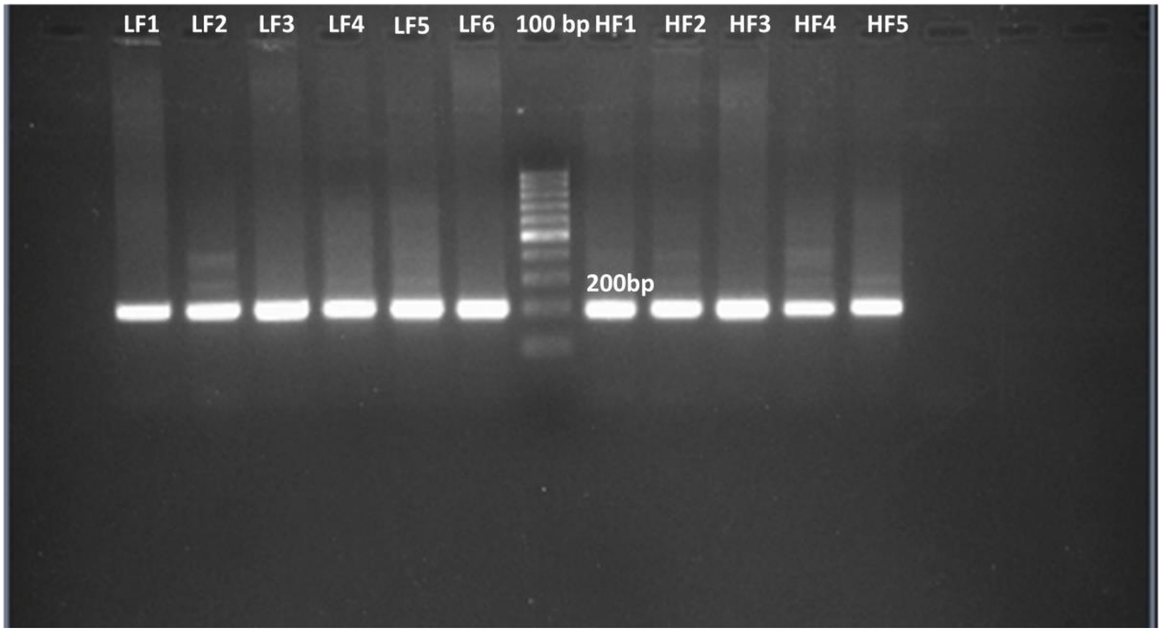

Supplementary Figure 6 (full view): The genomic DNA amplification of *Bos indicus x Bos taurusBBD129* gene. A) The genomic DNA PCR amplification of *BBD129* exon-1 from distinct fertility cross-bred bulls. B The genomic DNA PCR amplification of *BBD129* exon-2 has shown a band around 500bp in the high fertile cross-bred bulls. C) The genomic DNA PCR amplification of *BBD129* exon-2 has shown a band around 500bp in the low fertile cross-bred bulls Abbreviations: HF = High fertile crossbred bull, LF = Low fertile crossbred bull.

* Side wells are empty

Figure 5B

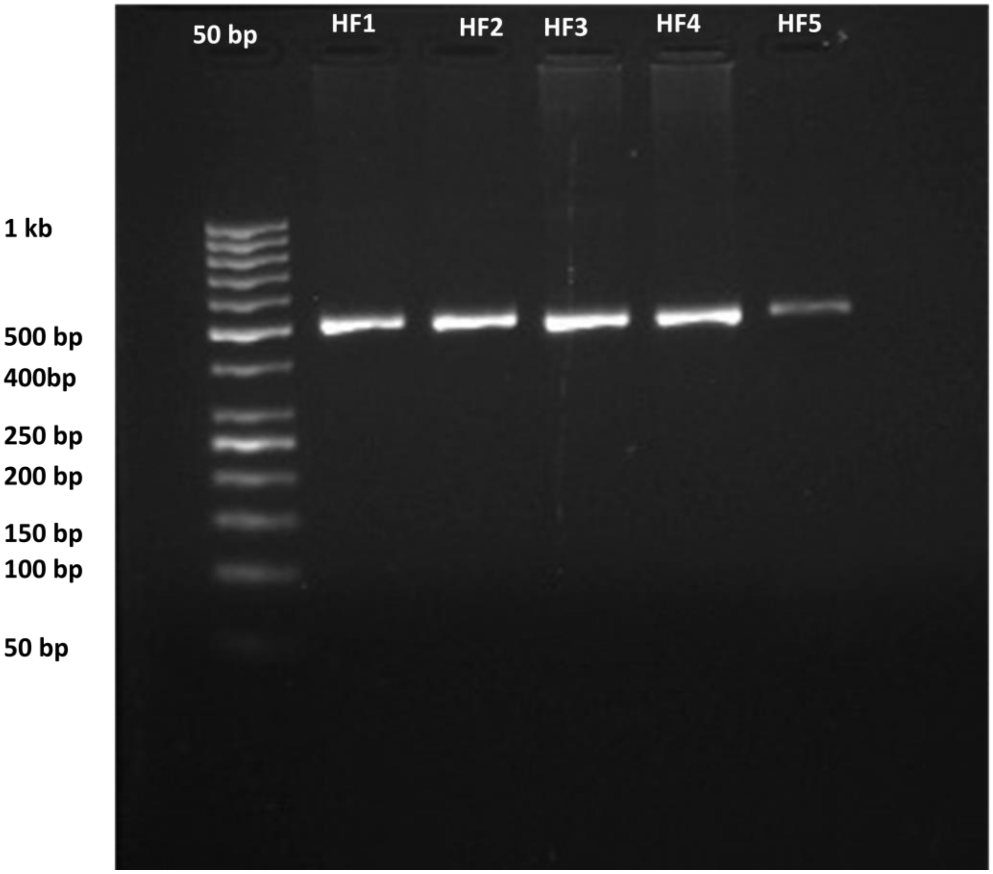

Supplementary Figure 6 (full view): The genomic DNA amplification of *Bos indicus x Bos taurusBBD129* gene. A) The genomic DNA PCR amplification of *BBD129* exon-1 from distinct fertility cross-bred bulls. B The genomic DNA PCR amplification of *BBD129* exon-2 has shown a band around 500bp in the high fertile cross-bred bulls. C) The genomic DNA PCR amplification of *BBD129* exon-2 has shown a band around 500bp in the low fertile cross-bred bulls Abbreviations: HF = High fertile crossbred bull, LF = Low fertile crossbred bull.

***** Last well is empty

Figure 5C

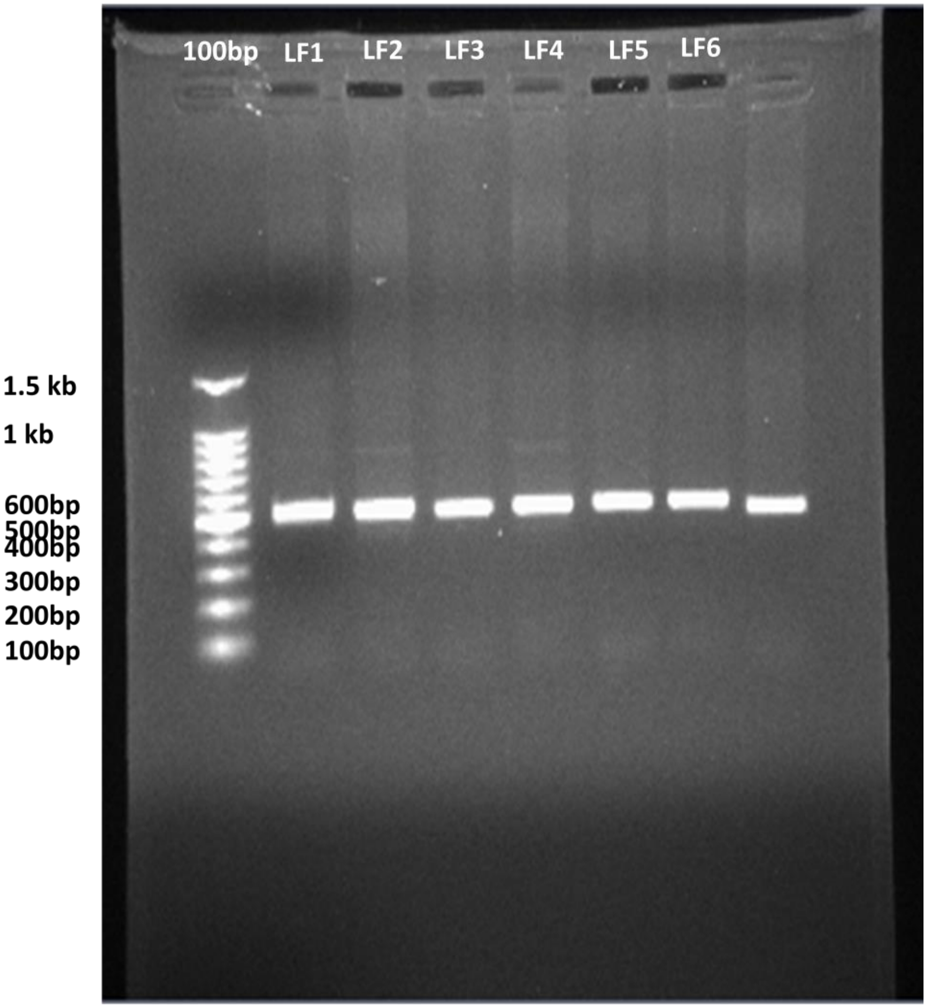

Supplementary figure 6 (full view): The genomic DNA amplification of *Bos indicus x Bos taurusBBD129* gene. A) The genomic DNA PCR amplification of *BBD129* exon-1 from distinct fertility cross-bred bulls. B The genomic DNA PCR amplification of *BBD129* exon-2 has shown a band around 500bp in the high fertile cross-bred bulls. C) The genomic DNA PCR amplification of *BBD129* exon-2 has shown a band around 500bp in the low fertile cross-bred bulls Abbreviations: HF = High fertile crossbred bull, LF = Low fertile crossbred bull.

* Last well was not of any interest.
