# Supplementary material for: Analysis of amplification and association polymorphisms in the bovine beta-defensin 129 (BBD129) gene revealed its function in bull fertility
Source: Sci Rep. 2022 Nov 9;12:19042. doi: 10.1038/s41598-022-23654-3 (PMC9646896; doi:10.1038/s41598-022-23654-3)
Supplement: Supplementary file 2 — Supplementary Information 2. [file 41598_2022_23654_MOESM2_ESM.pptx]

## Slide 1
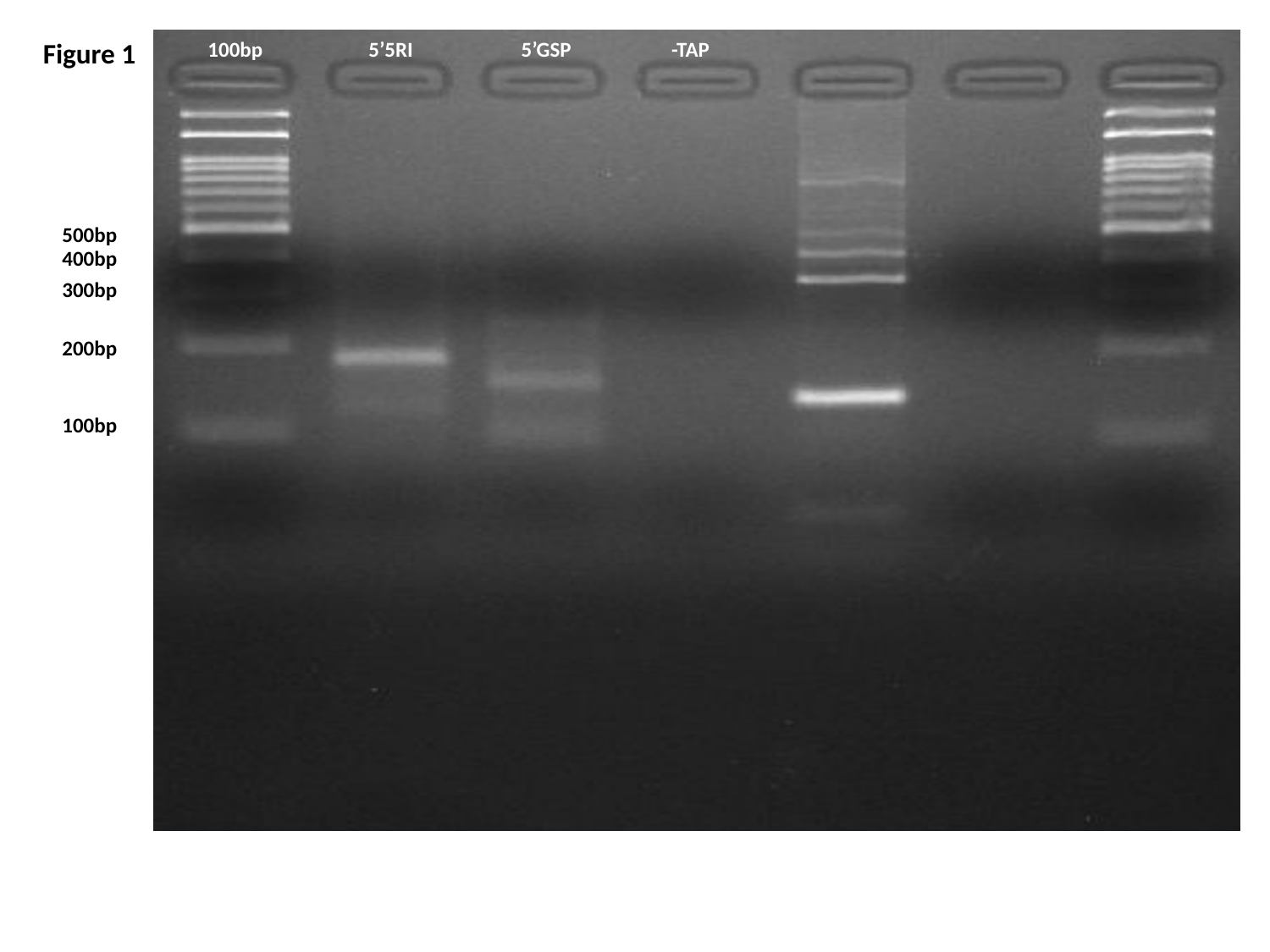

Figure 1
100bp
5’5RI
5’GSP
-TAP
500bp
400bp
300bp
200bp
100bp

## Slide 2
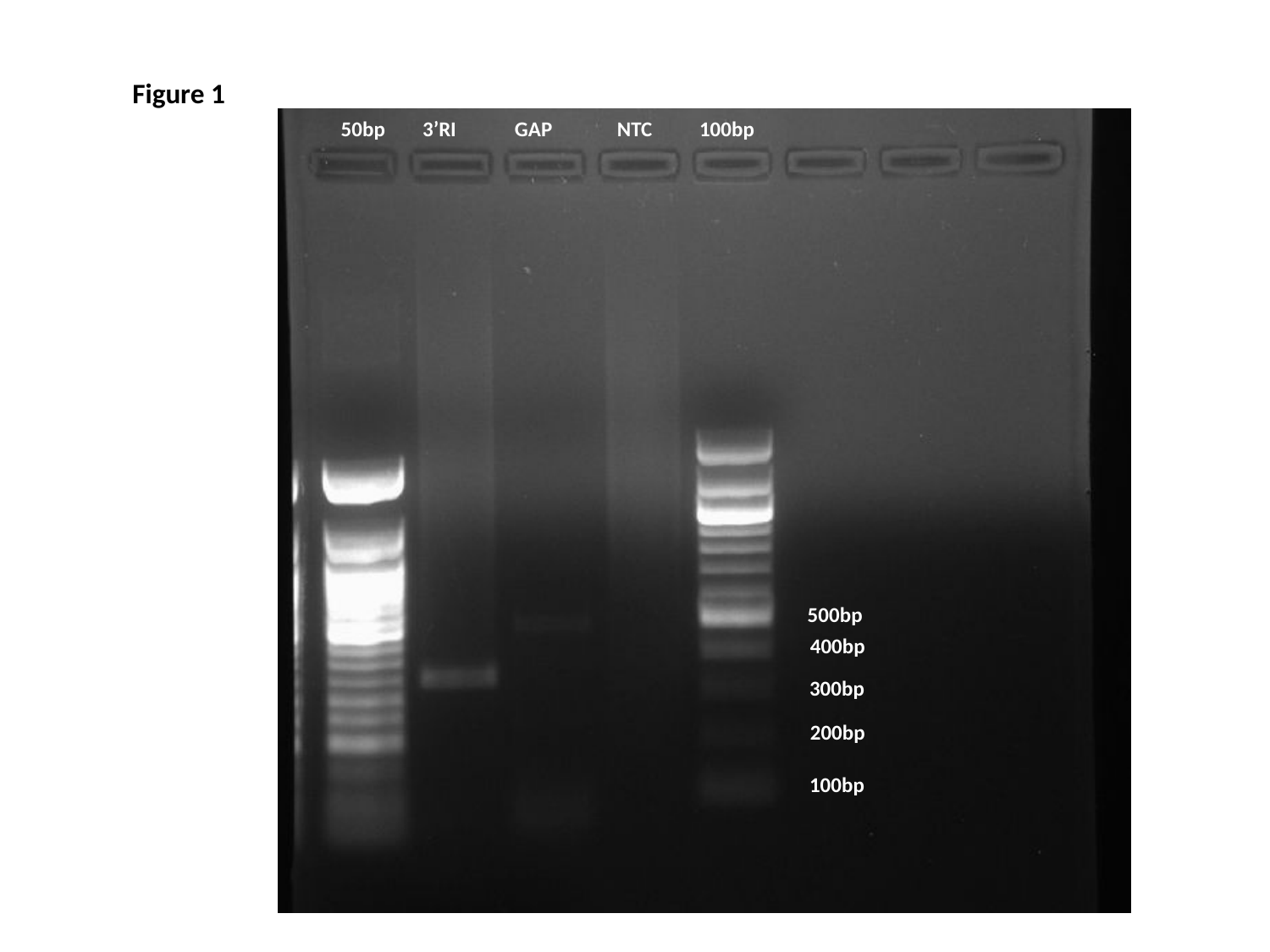

Figure 1
50bp
3’RI
GAP
NTC
100bp
500bp
400bp
300bp
200bp
100bp

## Slide 3
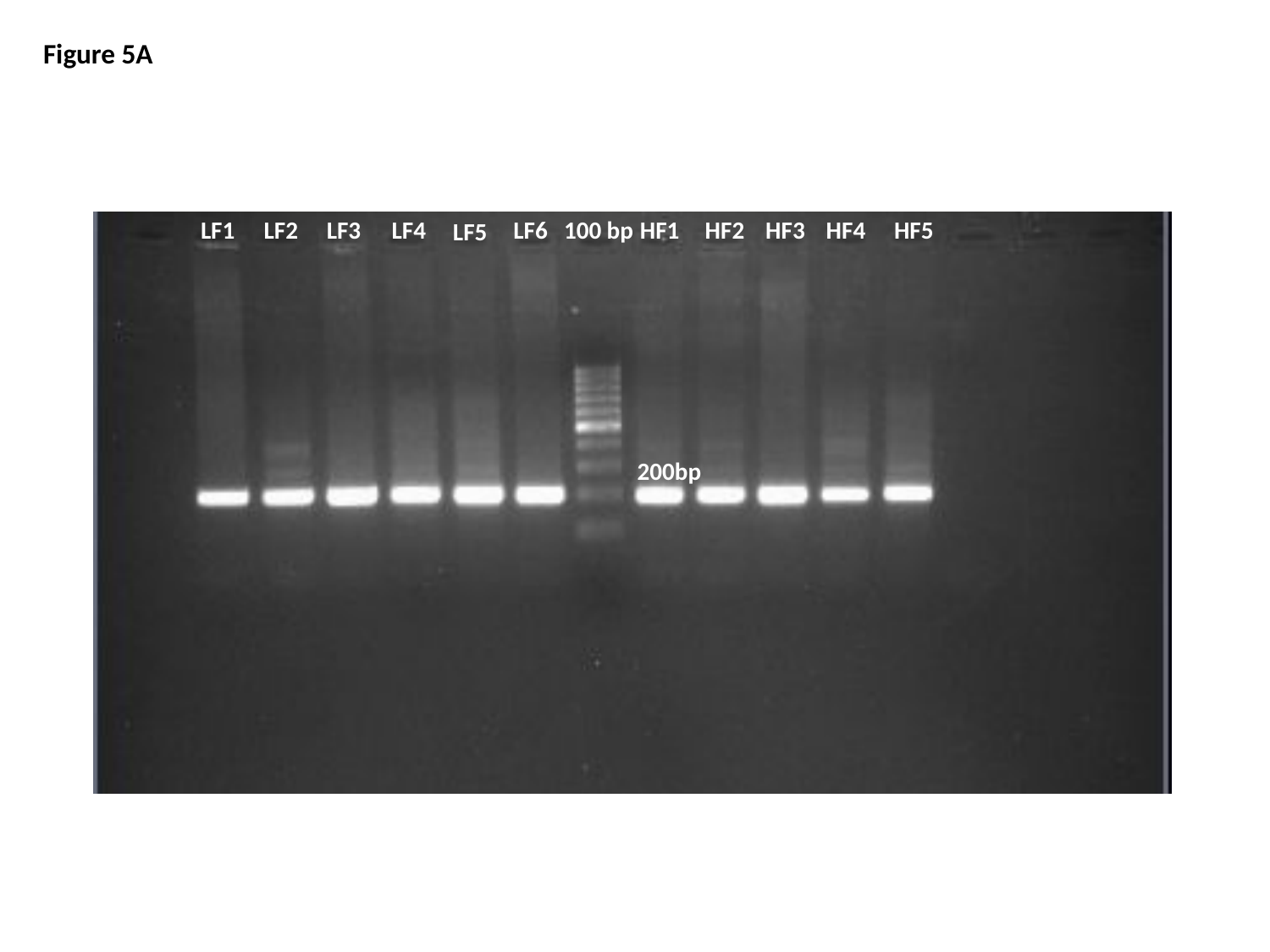

Figure 5A
LF1
LF2
LF3
LF4
LF6
100 bp
HF1
HF2
HF3
HF4
HF5
LF5
200bp

## Slide 4
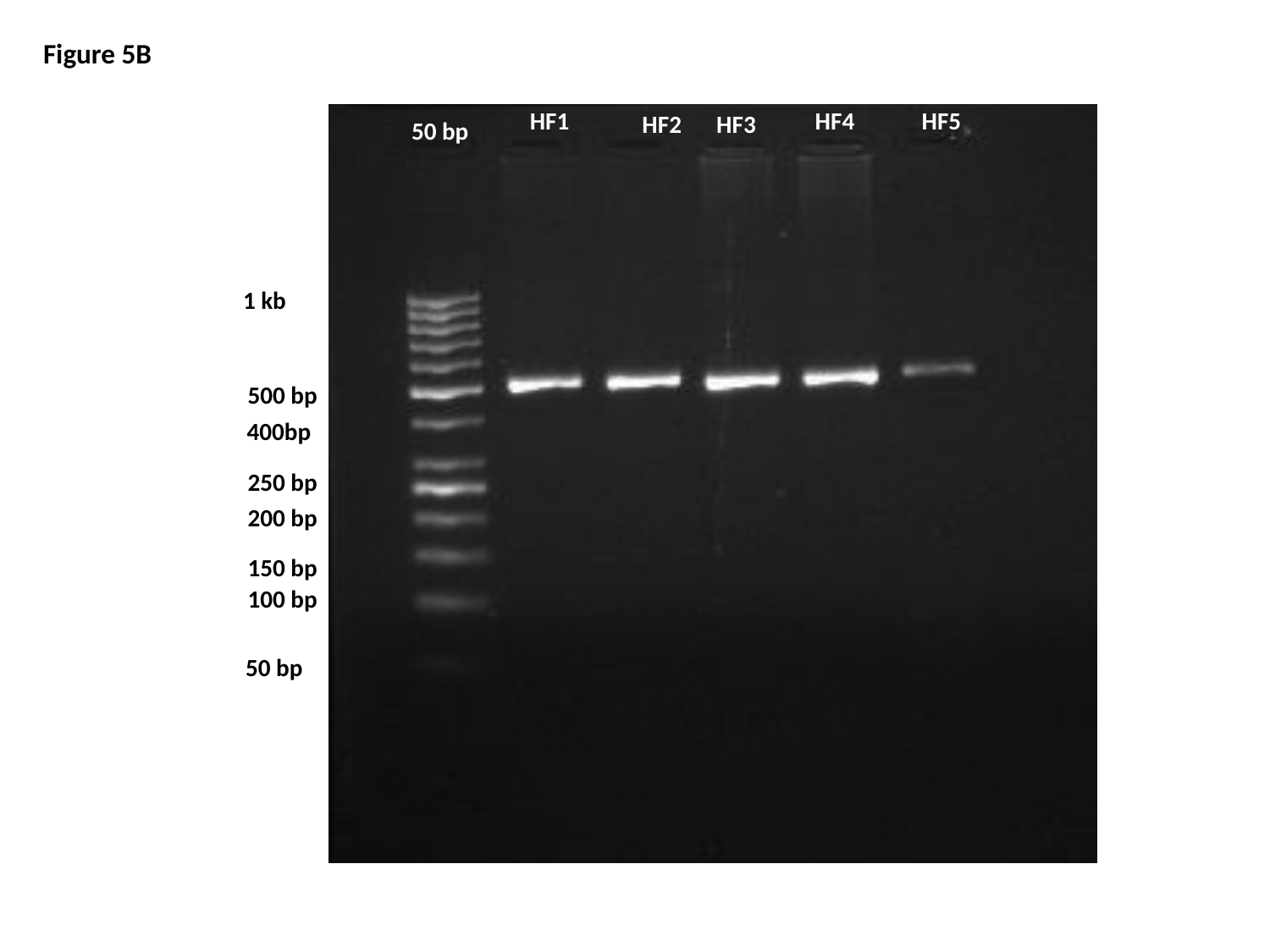

Figure 5B
50 bp
HF1
HF4
HF5
HF2
HF3
1 kb
500 bp
400bp
250 bp
200 bp
150 bp
100 bp
50 bp

## Slide 5
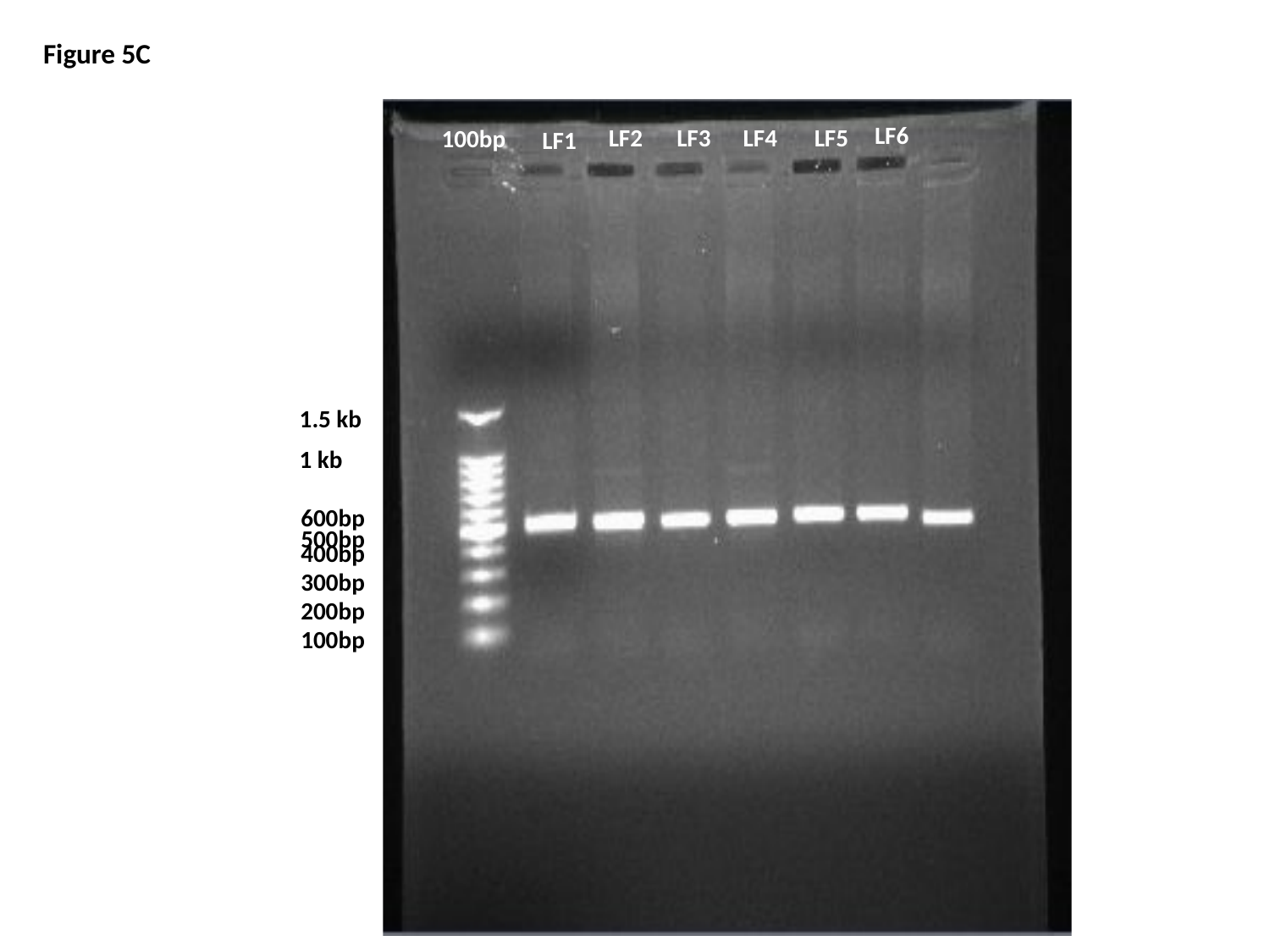

Figure 5C
LF6
LF2
LF3
LF4
LF5
100bp
LF1
1.5 kb
1 kb
600bp
500bp
400bp
300bp
200bp
100bp
